# Association between Toll-like receptor 9 signaling defect and developing post-infectious irritable bowel syndrome

**DOI:** 10.3389/fimmu.2025.1672117

**Published:** 2025-11-10

**Authors:** Satoshi Kotani, Yoshiyuki Mishima, Kenichi Kishimoto, Akihiko Oka, Naoki Oshima, Kousaku Kawashima, Kenjiro Matsumoto, Haruki Usuda, Koichiro Wada, Shunji Ishihara

**Affiliations:** 1 Department of Gastroenterology, Faculty of Medicine, Shimane University, Izumo, Japan; 2 Department of Internal Medicine II, Shimane University Faculty of Medicine, Izumo, Japan; 3 Department of Endoscopy, Shimane University Hospital, Izumo, Japan; 4 Laboratory of Pathophysiology, Doshisha Women’s College of Liberal Arts, Kyoto, Japan; 5 Department of Pharmacology, Faculty of Medicine, Shimane University, Izumo, Japan

**Keywords:** post-infectious irritable bowel syndrome, Citrobacter rodentium, Toll-like receptor 9, bradykinin receptor, R715, HOE 140

## Abstract

**Introduction:**

Post-infectious irritable bowel syndrome (PI-IBS) is a functional gastrointestinal disorder that develops after intestinal infection. A follow-up study after a waterborne outbreak of gastroenteritis indicated involvement of specific genetic variants including toll-like receptor (TLR)9, although its pathophysiological role remains unclear.

**Methods:**

To investigate the role of TLR9 in PI-IBS, *Citrobacter rodentium* was administered to wild-type (WT), and TLR2, 4, and 9 knockout (KO) mice. Six weeks after infection, visceral sensitivity was evaluated using barostat-based colorectal distention. Additional assessments include histological inflammation, intestinal permeability, gut microbiota, and colonic gene expression.

**Results:**

Only TLR9 KO mice developed significant visceral hyperalgesia despite findings indicating mild mucosal inflammation in the acute colitis phase and lack of persistent low-grade inflammation with hyperpermeability in the recovered phase. Microbiota analysis and fecal microbiota transfer demonstrated partial involvement of gut dysbiosis in PI-IBS development. Additionally, microarray, PCR, and immunohistochemistry findings showed that the expression levels of the bradykinin B1 and B2 receptors (BDKRB1 and BDKRB2) in colonic epithelium were significantly higher in infected TLR9 KO mice as compared to WT mice. Furthermore, administration of BDKRB1 antagonist R715 and BDKRB2 antagonist HOE 140 significantly suppressed visceral hyperalgesia.

**Conclusion:**

TLR9 deficiency leads to bradykinin receptor upregulation in the colonic epithelium following infectious colitis, contributing to the development of PI-IBS. Inhibition of these receptors alleviated visceral pain, indicating that bradykinin receptor antagonists may offer a novel therapeutic strategy for PI-IBS.

## 1 Introduction

Irritable bowel syndrome (IBS) is a functional gastrointestinal disorder characterized by chronic abdominal pain along with bowel movement disturbance, including diarrhea, constipation, or both (1). The global prevalence of IBS is approximately 10% in normal populations, though that varies largely depending on geographic factors and diagnostic criteria (2). Various investigations have been conducted to seek the cause of IBS from multiple perspectives, such as genetic predisposition, diet, mucosal inflammation, brain-gut-microbiota axis, stress, and anxiety (3–7). However, several details regarding the pathogenesis of IBS remain unclear and causal treatment is not currently available for clinical settings. Despite this being a nonfatal disorder, affected patients have significantly reduced quality of life (8) and the high prevalence of IBS has become a socioeconomic problem (9, 10). Thus, clarification of IBS pathogenesis and development of novel treatment strategies are considered to be urgent issues.

Some patients who previously had normal bowel habits develop IBS symptoms after acute gastroenteritis, a condition known as postinfectious IBS (PI-IBS) (11), with the diarrhea-dominant phenotype more commonly seen in PI-IBS cases (12, 13). *Campylobacter jejuni*, *Salmonella*, *Shigella*, and *Escherichia coli* are pathogens known to frequently cause PI-IBS in humans (14–17), while young age, female gender, psychological factors such as anxiety and depression, and severity of intestinal inflammation are thought to be risk factors for its development (18). Although details related to pathogenesis are not fully understood, a large number of clinical and basic studies suggest that PI-IBS is a multifactorial disorder, in which environmental factors such as infection can be a trigger in individuals possessing particular genetic variants (12).

As for genetics issues in PI-IBS cases, a follow-up study performed after a waterborne outbreak of gastroenteritis in Walkerton, Canada demonstrated that single nucleotide polymorphisms in Toll-like receptor (TLR)9, Interleukin (IL)-6, and Cadherin-1 were independent genetic risk factors for PI-IBS development (19). However, it has not been further clarified how these genetic mutations, especially TLR9, are involved in the pathogenesis of IBS. TLR9 is an innate immune-related receptor that recognizes unmethylated cytosine-phosphate-guanosine (CpG)-DNA from bacteria and viruses (20). Even though CpG-DNA is scarce in mammals and mostly methylated, TLR9 can recognize microbial-specific unmethylated CpG-DNA in the human body and abnormal response targeting of self-DNA by TLR9 can trigger development of autoimmune diseases, such as psoriasis, autoimmune arthritis, and ulcerative colitis (21–24). However, to the best of our knowledge, there is no study available that investigated in detail TLR9 signaling in functional intestinal diseases including IBS. The present investigation was conducted to examine the role of TLR9 signaling in the pathogenesis of PI-IBS and develop new IBS therapeutic strategies.

## 2 Materials and methods

### 2.1 Animals

C57BL/6J WT mice were purchased from Charles River Laboratories Japan (Yokohama, Kanagawa, Japan), and TLR2, TLR4, and TLR9 KO mice from Oriental Bio Service (Kyoto, Japan). Mice were bred under specific pathogen-free conditions at the animal facility of Shimane University School of Medicine, then maintained in plastic cages at 20-22°C with a 12-hour light/dark cycle, and provided with food and water. Eight- to nine-week-old mice were used in the experiments. Mice were euthanized by carbon dioxide (CO_2_) inhalation using the gradual-fill (displacement) method (100% CO_2_; 40% of chamber volume per minute), in accordance with the 2020 AVMA Guidelines for the Euthanasia of Animals. Unconsciousness was confirmed by loss of righting reflex; flow was maintained for 5 min after respiratory arrest, and death was ensured by cervical dislocation.

### 2.2 C. rodentium infection

*C. rodentium* (DBS100, 51459™, ATCC, Manassas, Virginia, USA) was cultured overnight in medium composed of 5 mL of Luria-Bertani (LB) broth (Becton, Dickinson and Company, Franklin Lakes, New Jersey, USA) at 37°C, with rotation at 150 rpm. Sixteen hours later, 1 mL was obtained and added to 99 mL of fresh LB medium (Becton, Dickinson and Company), then incubated for another four hours. After centrifugation at 2, 500 rpm for 10 minutes, phosphate-buffered saline (PBS) was added to dissolve the pellets, resulting in 5.0×10^9^ colony forming units (CFU)/mL. Mice were administered 1.0×10^9^ CFU (200 μL) *of C. rodentium* or the same amount of PBS using oral gavage.

### 2.3 Evaluation of VMR to colorectal distention with rectal balloon dilation

Five weeks after infection, mice were anesthetized intraperitoneally using medetomidine hydrochloride at 0.3 mg/kg, midazolam at 4 mg/kg, and butorphanol tartrate at 5 mg/kg, and electrode implantation in the abdominal wall was performed. Measurements of VMR to colorectal distention were performed one week later, with the animal held in a mouse holder to prevent movement and the balloon placed 5 mm from the anus. Then, 10-second distention was performed three times with one-minute intervals in each mouse at four different levels of balloon pressure (15, 30, 45, and 60 mmHg) controlled by use of a Distender Series IIR Dual Balloon Barostat System (G&J Electronics, Toronto, Ontario, Canada). Obtained data was analyzed with the Analyze II software package (Starmedical, Tokyo, Japan). Values for electromyographic activity above the baseline value were obtained, with each value noted with balloon dilation subtracted from that without dilation. Three values were obtained at each pressure level, with median values used for statistical analysis. The bradykinin B1 receptor antagonist R715 was obtained from MedChemExpress (Monmouth Junction, New Jersey, USA; Cat. No. HY-103290). The bradykinin B2 receptor antagonist HOE 140 (icatibant) was obtained from TOCRIS, part of Bio-Techne (Bristol, UK; Cat. No. 3014; purchased via Funakoshi, Tokyo, Japan). Solutions were prepared fresh on the day of use according to the manufacturers’ datasheets.

### 2.4 Histological analysis of mouse colons

Following assessment of VMR, the mice were euthanized. The distal colon was removed and fixed with 10% neutral buffered formalin, then tissue sections were stained with hematoxylin and eosin. Histological damage score included severity of epithelial damage (0-3), degree of inflammatory cell infiltration (0-3), and presence or absence of goblet cell depletion (0-1). Crypt length measurements were obtained as the mean of 10 well-oriented crypts from all mice. The histological evaluations were evaluated in a blinded manner (25–27).

### 2.5 Evaluation of colonic inflammation using reverse transcription polymerase chain reaction

Total RNA was isolated from the distal colon using an RNeasy Micro Kit (QIAGEN, Venlo, Nederland). First-strand complementary DNA was synthesized from 1 µg of total RNA using M-MLV Reverse Transcriptase (Invitrogen, Waltham, Massachusetts, USA), according to the manufacturer’s instructions. Quantitative reverse-transcription polymerase chain reaction examinations were performed with a Mastercycler EP realplex 2S system (Eppendorf, Hamburg, Germany) using SYBR Green quantitative PCR SuperMix (Invitrogen, Waltham, Massachusetts, USA) to quantify gene expression. The following PCR primers were used in this study (28–30). *Il1b*-F GAAATGCCACCTTTTGACAGTG and *Il1b*-R TGGATGCTCTCATCAGGACAG; *Il6*-F CTGCAAGAGACTTCCATCCAG and *Il6*-R AGTGGTATAGACAGGTCTGTTGG; *Tnfa*-F ACCCTCACACTCAGATCATCTTCTC and *Tnfa*-R TGAGATCCATGCCGTTGG; *Il10*-F GTCATCGATTTCTCCCCTGTG and *Il10*-R CCTTGTAGACACCTTGGTCTTGG; *Bdkrb1*-F CCCCTCCCAACATCACCTC and *Bdkrb1*-R GGACAGGACTAAAAGGTTCCCC; *Bdkrb2*-F GGGTTTCTGTCGGTGCATGA and *Bdkrb2*-R TTGTGTGGTGACGTTGAACAT; *Gapdh*-F GGTCGGTGTGAACGGATTTG and *Gapdh*-R TGTAGACCATGTAGTTGAGGTCA. The results are expressed as relative to the housekeeping gene *Gapdh*.

### 2.6 Microarray analysis

Total RNA was prepared from distal colon samples obtained from *C. rodentium*-treated WT and TLR9 KO mice as described above. RNA samples were sent to Filgen (Aichi, Japan), where DNA microarray analysis was performed as previously described (29). Based on the results of altered gene expression, pathway analysis was performed with a Microarray Data Analysis Tool Ver.3.2 (Filgen).

### 2.7 Determination of intestinal permeability

FITC-dextran with a molecular weight of 4 kDa (Chondrex, Woodinville, Washington, USA) was used to evaluate intestinal permeability. Food was not given for four hours, then the mice were orally administered 20 mL/kg FITC-dextran and fasting was continued for three hours. Following euthanasia, blood was obtained from the right atrium. Blood samples were centrifuged at 10, 000 rpm for 10 minutes to collect plasma and fluorescence was measured with a GloMax^®^ Discover Microplate Reader (Promega Corporation, Madison, Wisconsin, USA) using 96-well plates with excitation at 475 nm and emission at 500–550 nm. FITC-dextran concentrations were calculated with a standard concentration curve ranging from 0 to 12.5 µg/mL.

### 2.8 Immunohistochemistry

Immunohistochemical staining was performed as previously described (31). The primary antibodies used were rabbit anti-Bdkrb1 (1:1000, Bioss, Boston, MA, USA, BS8675R), rabbit anti-Bdkrb2 (1:1000, Bioss, Boston, MA, USA, BS2422R), guinea-pig anti-keratin8/18 (1:3000, Progen Biotechnik, Heidelberg, Germany, GP11), and Alexa Fluor 647 rabbit PGP9.5 (1:200, Abcam, Cambridge, UK, AB_196173). The secondary antibodies were Alexa Fluor 488 donkey anti-rabbit IgG (1:800, Life Technologies, Carlsbad, CA, USA) and Alexa Fluor 594 donkey anti-guinea pig (1:800, Jackson ImmunoResearch Inc., West Grove, PA, USA). Stained tissues were observed using a confocal microscope (LSM800; Zeiss, Oberkochen, Germany).

### 2.9 Fecal bacteria analysis

Bacterial DNA was extracted from stool samples using a NucleoSpin^®^ DNA Stool kit (MACHEREY-NAGEL GmbH & Co. KG, Dueren, Germany), according to the manufacturer’s instructions, and stored at −80°C until use. The V3–V4 region of bacterial 16S rRNA was amplified by PCR using specific primers with the following sequences: forward primer, 5′-TCGTCGGCAGCGTCAGATGTGTATAAGAGACAGCCTACGGGNGGCWGCAG-3′; reverse primer, 5′-GTCTCGTGGGCTCGGAGATGTGTATAAGAGACAGGACTACHVGGGTATCTAATCC-3′. The amplicon was purified with AMPure XP beads, then a barcode sequence was added to each amplicon using an Illumina Nextera XT Index kit, ver. 2 (Illumina, San Diego, California, USA) for labeling and to distinguish the samples. The barcoded library was purified as described above, then diluted to 4 nmol/L in 10 mmol/L of Tris-HCl (pH 8.0). Five microliters of each diluted sample was pooled and then further diluted to 6 pmol/L using buffer from the respective sequencing kit. This sample DNA library was applied to an MiSeq Reagent kit, ver. 3 (Illumina) and sequenced with a 2×300-bp paired end using the kit and spiked with 5% PhiX control DNA (6 pmol/L). Annotation and calculation of obtained sequences were processed using 16S Metagenomics Database Creator, ver. 1.0.0.

### 2.10 Fecal microbiota transplantation

Donor feces were obtained from TLR9 KO mice at six weeks after *C. rodentium* infection, then stored at -80°C until fecal microbiota transplantation (FMT). Prior to FMT, gut microbiota in recipient was depleted using a three-day treatment with a broad-spectrum antibiotic cocktail, including oral administration of vancomycin (100 µL, 5 mg/mL) and metronidazole (100 µL, 10 mg/mL), as well as supplementation of drinking water with ampicillin (1 g/L) and neomycin (0.5 g/L), as previously described (32–34). FMT was performed one day after completion of antibiotic treatment,. Frozen stool samples were suspended in PBS at a ratio of 15 mL/gram of feces, then 200 μL of fecal slurry was administered twice into each recipient mouse by oral gavage, with a 72-hour interval between administrations.

### 2.11 Statistics

Statistical analyses were performed with GraphPad Prism 9 (GraphPad Software, San Diego, California, USA). Student’s t test was used to compare means of two groups, and one-way or two-way ANOVA to compare means of multiple groups. Tukey’s and Holm-Sidak’s multiple comparisons testing was conducted for *post hoc* analysis. The level of statistical significance was set at *p* < 0.05.

## 3 Results

### 3.1 Citrobacter rodentium induces colonic inflammation in acute phase

**Figure 1:**
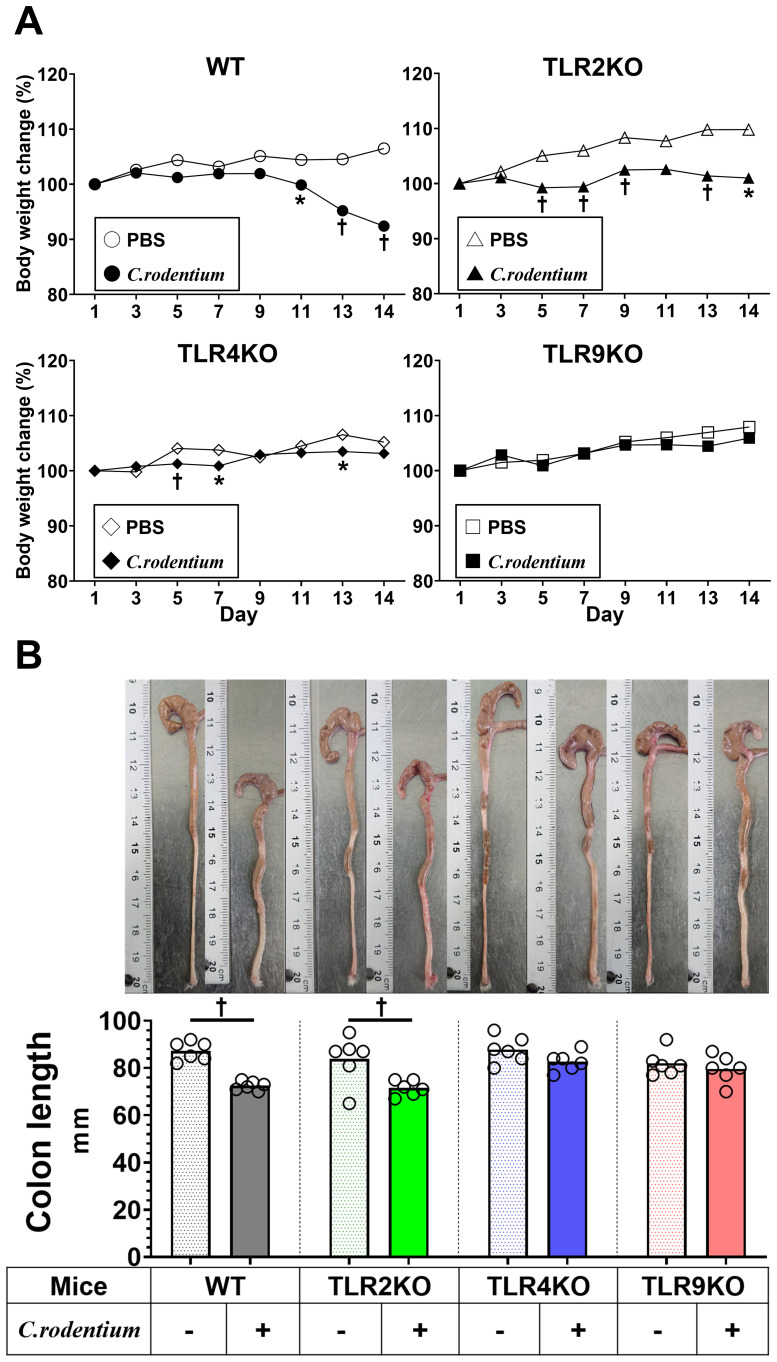
*C. rodentium* induced acute colitis within two weeks. *C. rodentium* (1.0×10^9^ colony forming units) or phosphate-buffered saline (PBS) was administered to wild-type (WT), Toll-like receptor (TLR)2 knockout (KO), TLR4 KO, and TLR9 KO mice (n=6/group) on day 1. **(A)** Body weight was measured every other day. **(B)** Mice were euthanized 14 days after *C. rodentium* infection and colon length measured. Values were obtained using Student’s t test and are presented as the mean. *p <0.05, ^†^p <0.01, as compared with PBS group.

**Figure 2:**
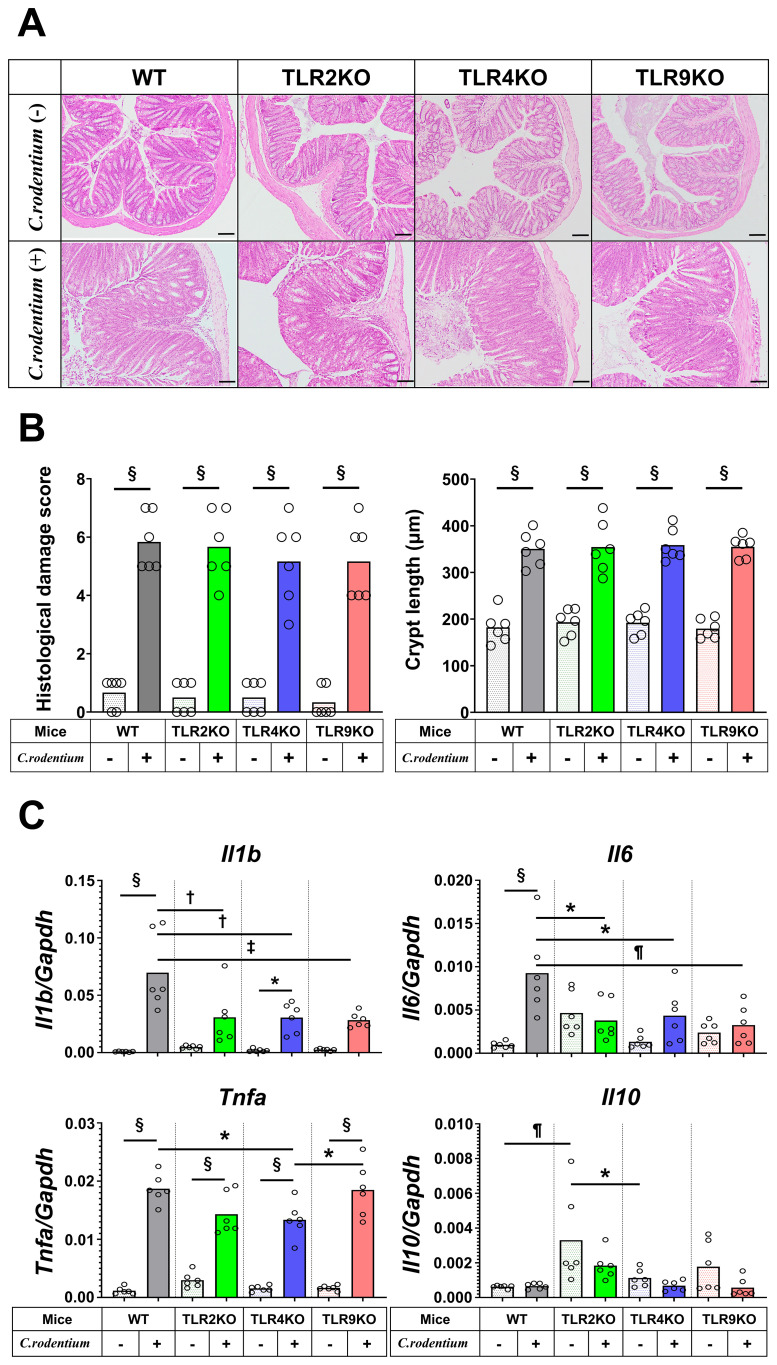
Histological analysis and cytokine profiles of *C. rodentium*-infected mice in acute phase. *C. rodentium* or PBS was administered to WT, TLR2 KO, TLR4 KO, and TLR9 KO mice (n=6/group), then colon assessment was performed 14 days after infection. **(A)** Histological analysis of distal colon sections. Hematoxylin-eosin staining; original magnification: ×100. Scale bar = 100 μm. **(B)** Histological damage score and crypt length were determined on day 14. **(C)** RT-PCR assays for *Il1b*, *Il6*, *Tnfa*, and *Il10* were performed using distal colon tissues, then obtained gene expression values were normalized based on *Gapdh*. Values were obtained using a one-way ANOVA test and are presented as the mean. *p <0.05, ^†^p <0.01, ^¶^p <0.005, ^‡^p <0.001, ^§^p <0.0001. Tukey’s multiple comparisons test was used for *post hoc* analysis.

A previous clinical follow-up study suggested that TLR9 dysfunction is a potential mechanism involved in development PI-IBS (19), thus we sought to determine the role of TLR9 signaling in mice with and after resolution of infectious colitis. Acute colitis was induced in wild-type (WT), TLR2 knockout (KO), TLR4 KO, and TLR9 KO mice by administrating the mouse pathogen *C. rodentium*, then colitis severity was evaluated based on body weight changes, pathology, and mucosal cytokine gene expression at two weeks after infection (acute phase). All mice without *C. rodentium* infection steadily gained body weight, while those administered the pathogen showed various body weight changes dependent on the absence of a particular TLR, with severe, moderate, and mild weight loss noted in the WT, TLR2 KO, and TLR4 KO groups, respectively (**Figure 1A**). On the other hand, TLR9 KO mice did not show significant body weight loss following *C. rodentium* infection (**Figure 1A**). Consistent with the effects on body weight, colons from *C. rodentium*-infected WT and TLR2 KO mice were significantly shortened and thick, whereas infected TLR4 KO and TLR9 KO mice showed only mild shortening and thickness (**Figure 1B**). Histological findings indicated that *C. rodentium* infection induced massive inflammatory cell infiltration with marked edema and colonic hyperplasia, which resulted in increased crypt length, in the colons of the examined mouse types (**Figure 2A**), while histological damage score and crypt length were not different among any of those infected with *C. rodentium* (**Figure 2B**). Furthermore, RT-PCR assay findings showed significantly increased proinflammatory cytokine gene expressions in the colons of all mice, especially the WT group (**Figure 2C**). These results indicate that *C. rodentium* induces acute colitis in all types of mice, though the TLR9 KO group appeared to have milder mucosal inflammation as compared to the others. Although Dunlop et al. demonstrated that intestinal hyperpermeability is associated with deterioration of colitis and development of IBS symptoms (35), in the present study, *C. rodentium* infection did not increase mucosal permeability in TLR9 KO mice or the other types of mice examined (**Supplementary Figure S1**).

### 3.2 TLR9-deficient mice develop PI-IBS after recovery from C. rodentium infection

**Figure 3:**
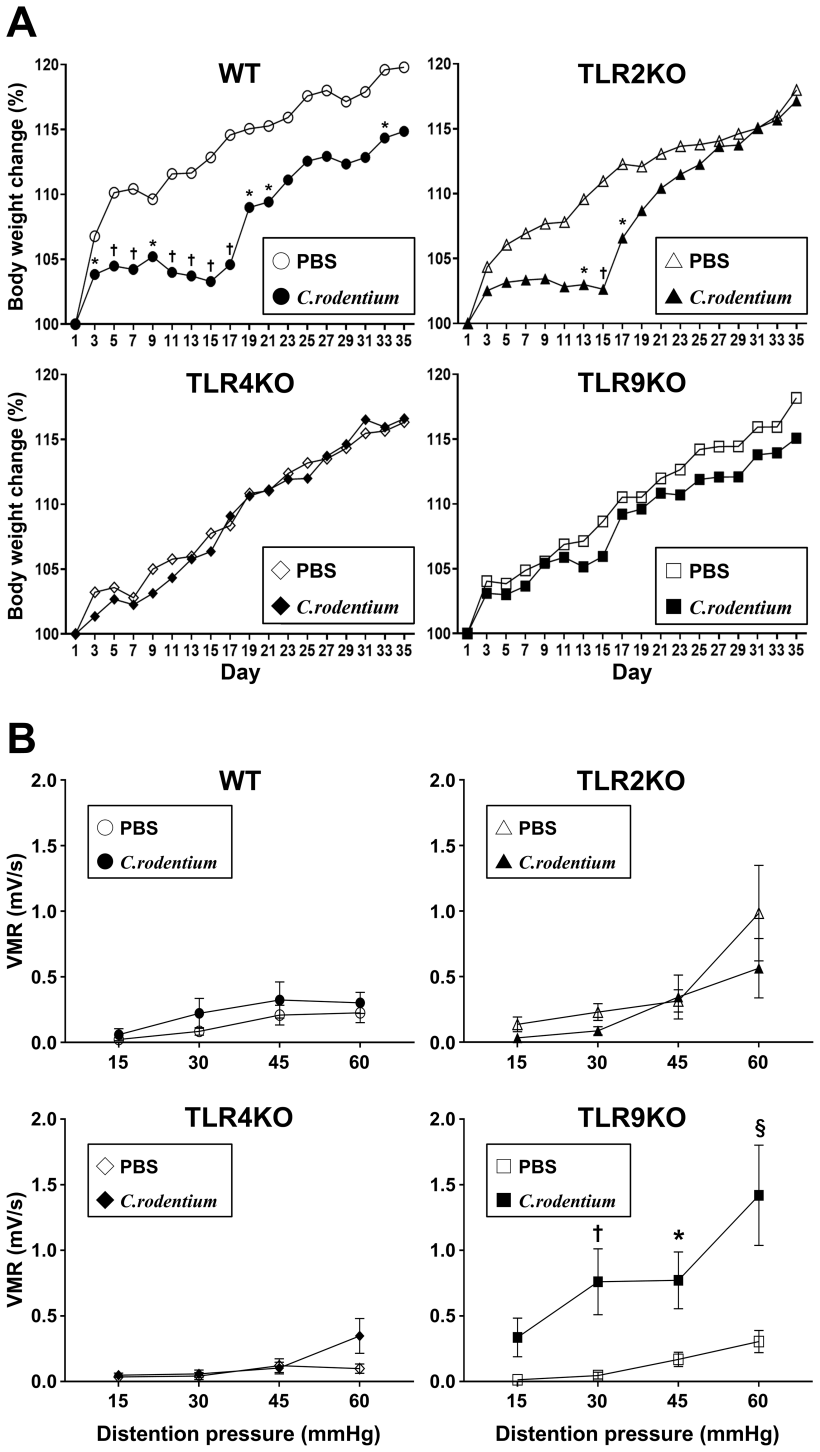
*C. rodentium* induced visceral sensitivity in *TLR9 KO mice*. *C. rodentium* or PBS was administered to WT, TLR2 KO, TLR4 KO, and TLR9 KO mice (n=12/group) on day 1. **(A)** Body weight was measured every other day until the endpoint (day 35). Values were obtained using Student’s t test and are presented as the mean. *p <0.05, ^†^p <0.01, as compared with PBS group. **(B)** Five weeks after infection, mice were anesthetized and electrodes implanted in the abdominal wall, then evaluation of visceromotor response (VMR) to colorectal distention was performed at six weeks after infection. Four different levels of pressure (15, 30, 45, and 60 mmHg) were used for balloon dilation in each mouse. A 10-second distention was performed three times with one-minute intervals at each pressure and the median value used. Values were obtained using a two-way ANOVA test and are presented as the mean ± SEM. *p <0.05, ^†^p <0.01, ^§^p <0.0001, as compared with PBS group. Tukey’s multiple comparisons test was used for *post hoc* analysis.

Previous reports have noted that *C. rodentium*-induced acute colitis was totally recovered within 21–28 days with spontaneous elimination of *C. rodentium* in the presence of normal mucosal immunity (36). Thus, we conducted a parallel study to evaluate IBS features at six weeks after infection when the mice had fully recovered from *C. rodentium*-induced colitis. Consistent with the aforementioned body weight changes, *C. rodentium*-infected WT and TLR2 KO mice showed poor weight gain during the first two weeks after infection, which then rapidly recovered after the acute colitis phase (**Figure 3A**), while neither TLR4 KO nor TLR9 KO mice showed body weight loss throughout the observation period. Importantly, there was no significant difference regarding final body weight ratio among the groups of infected mice after five weeks (recovered phase) (**Figure 3A**). Next, visceral sensitivity in the mice after six weeks was examined with use of a barostat, which allowed for quantitative assessments of the severity of IBS features. Interestingly, only TLR9 KO mice infected with *C. rodentium* developed significant visceral hyperalgesia (**Figure 3B**, **Supplementary Figures S2**, **S3**), while TLR2 KO and TLR4 KO, as well as WT mice did not show visceral hypersensitivity even after resolution of *C. rodentium* infection (**Figure 3B**). Additionally, there was no difference noted for *C. rodentium*-induced visceral hypersensitivity in TLR9 KO mice based on gender (**Supplementary Figure S3**). Together, these results indicate that *C. rodentium* can induce PI-IBS in the absence of signaling by the TLR9, but not in the absence by that of TLR2 or 4, which does not appear to depend on the severity of acute inflammation.

### 3.3 Mechanistic insights into pathogenesis of PI-IBS in TLR9 KO mice

**Figure 4:**
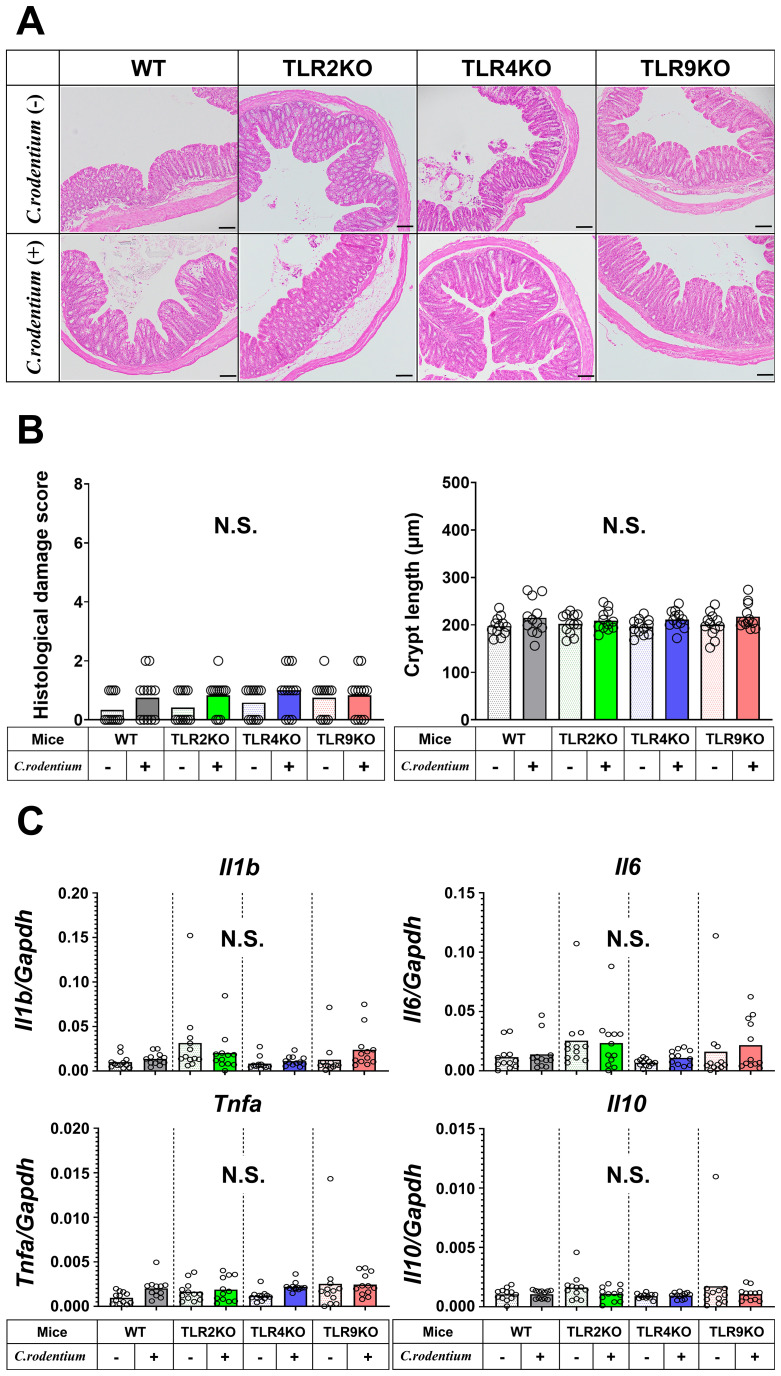
Histological analysis and cytokine profiles of *C. rodentium*-infected mice in recovered phase. **(A)** Distal colons from WT, TLR2 KO, TLR4 KO, and TLR9 KO mice with or without *C. rodentium* infection (n=12/group) were evaluated histologically on week six. Hematoxylin-eosin staining; original magnification: ×100. Scale bar = 100 μm. **(B)** Histological damage scores and crypt length of distal colon specimens. **(C)** RT-PCR assays for *Il1b*, *Il6*, *Tnfa*, and *Il10* were performed using the distal colon tissues and the values of gene expression were normalized based on *Gapdh*. Values were obtained with a one-way ANOVA test and are presented as the mean. N.S., not significant.

Previous reports have suggested that persistent low-grade mucosal inflammation, intestinal hyperpermeability, and changes in intestinal microbiota (dysbiosis) are key factors for development of PI-IBS (13, 17, 35, 37–41), thus these potential mechanisms were further investigated in mice with or without PI-IBS in the present study. Histological damage scores and crypt length measurements showed that severe colitis seen after two weeks was ameliorated in all samples obtained after six weeks, while levels of mucosal inflammation were all similar regardless of the TLR status in the mice (**Figures 4A, B**). Similarly, as compared with samples obtained after two weeks (**Figure 2C**), both proinflammatory and regulatory cytokine gene levels in colons of *C. rodentium*-infected mice were downregulated and had returned to basal levels, while the mucosal cytokine profile was also not significantly different among any of the TLR mutation types (**Figure 4C**). Moreover, the FITC-dextran assay results demonstrated that *C. rodentium*-treated TLR9 KO as well as the other infected mice did not have increased intestinal permeability (**Supplementary Figure S4**).

As for dysbiosis following *C. rodentium* infection, gut microbiota from all groups of mice at six weeks after infection demonstrated similar findings, with no significant differences at the phylum level noted (**Supplementary Figure S5**). On the other hand, the proportions of *Clostridiaceae_1* at the family level and *Clostridium_sensu_stricto* at the genus level were greater in TLR9 KO mice as compared to those in the other types of mice (**Supplementary Figures S6A-D**). We also confirmed that *C. rodentium* was not detected at the species level in any mice at the six-week timepoint. To further investigate whether dysbiosis is a cause or consequence of PI-IBS, microbiota transplantation (FMT) was performed using feces from infected TLR9 KO mice (**Supplementary Figure S7**). Those stool samples induced a modest increase in VMR in response to colorectal distention in TLR9 KO mice, but not in WT mice, at a distention pressure of 60 mmHg, though the difference did not reach statistical significance (**Supplementary Figure S8A**). Together, these gut microbial analyses indicate that intestinal dysbiosis is partially implicated as a causal factor in development of PI-IBS in *C. rodentium*-treated TLR9 KO mice.

### 3.4 Bradykinin receptors upregulated in C. rodentium-infected TLR9 KO mice

**Figure 5:**
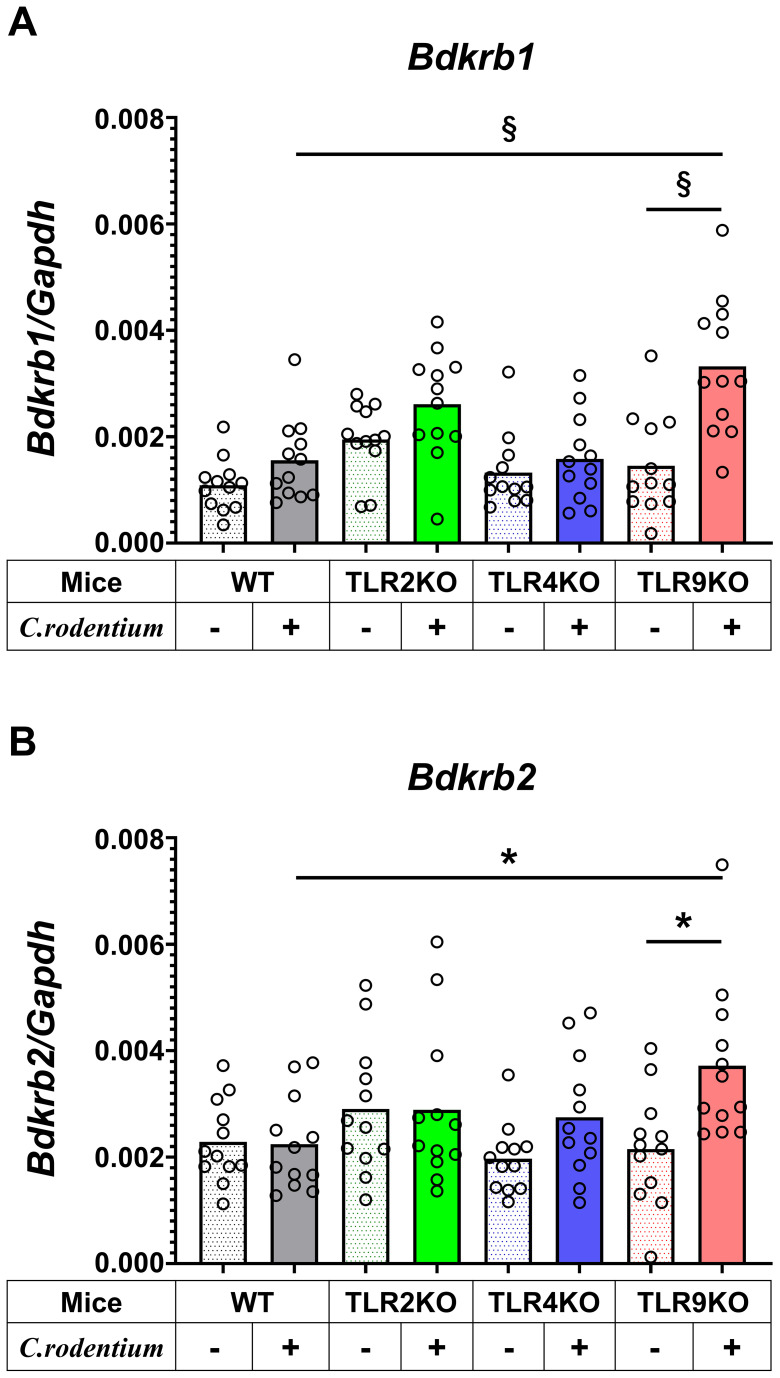
Bradykinin receptors upregulated in *C. rodentium*-infected TLR9 KO mice. Expression levels of **(A)** bradykinin B1 receptor (*Bdkrb1*) and **(B)** B2 receptor (*Bdkrb2*) in distal colons obtained from WT, TLR2KO, TLR4KO, and TLR9 KO mice with and without *C. rodentium* infection (n=12/group) were assessed by RT-PCR at six weeks after infection. Values were obtained with a one-way ANOVA test and are presented as the mean. *p <0.05, §p <0.0001, as compared with PBS group. Holm-Sidak’s multiple comparisons test was used for post hoc analysis.

**Figure 6:**
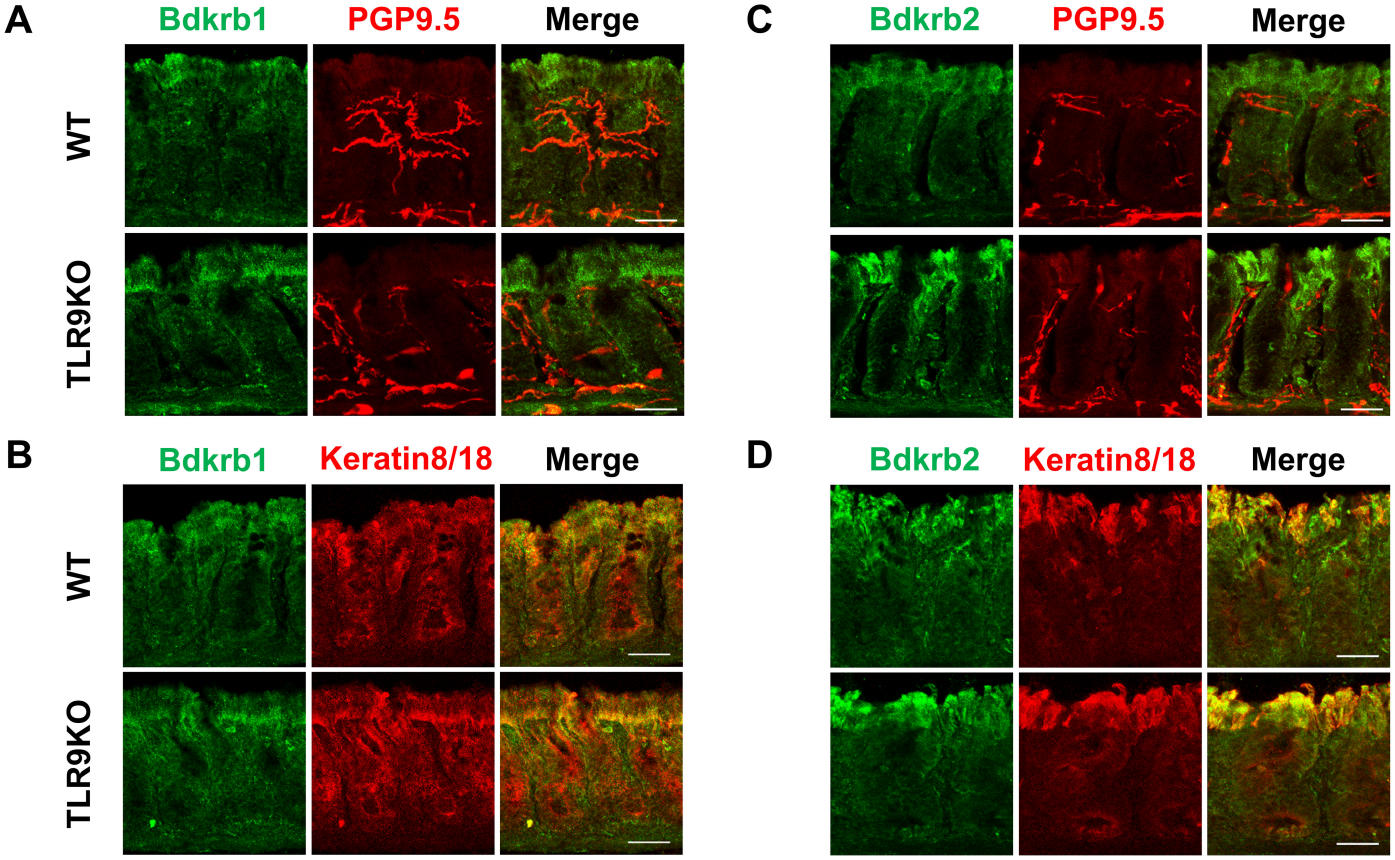
Bdkrb1 and Bdkrb2 expressed in intestinal mucosal epithelium but not enteric nervous system. Immunofluorescence staining was performed with distal colons from *C. rodentium*-treated WT or TLR9 KO mice at six weeks after infection. **(A)** Bdkrb1 and PGP9.5. **(B)** Bdkrb1 and Keratin8/18. **(C)** Bdkrb2 and PGP9.5. **(D)** Bdkrb2 and Keratin8/18. Original magnification: ×200. Scale bar = 20 μm.

The molecular mechanism was further examined by microarray analysis in colon samples from *C. rodentium*-infected WT and TLR9 KO mice, which revealed significant differences between those mice for several pathways (**Table 1**). Among the altered pathways, we focused on peptide G protein-coupled receptors (GPCRs), as those represent important pathways in pain sensation. Moreover, upregulation of the *Bdkrb2* gene in the peptide GPCRs pathway is known to be involved in the pathogenesis of visceral hyperalgesia (42–44). Bradykinin is produced in the kinin-kallikrein system in plasma and tissues, which has a variety of physiological functions including circulation regulation, vasodilation, edema, inflammation, and pain (45). Bradykinin receptor, a G-protein-coupled receptor, is expressed in nociceptors, macrophages, fibroblasts, and mast cells. Bradykinin receptor B1 (Bdkrb1) is upregulated during inflammation, while bradykinin receptor B2 (Bdkrb2) is homeostatically expressed and normally induces physiological effects (46). Bdkrb2 is involved in the pathogenesis of hereditary angioedema, with affected individuals showing severe abdominal pain as well as swelling of the skin. In the present study, expression levels of bradykinin receptors in intestinal tissues were examined using RT-PCR assay. *Bdkrb2* was not upregulated by *C. rodentium* infection in any of the mice groups in the acute phase, while only TLR9 KO mice showed an increase in *Bdkrb2* expression in the recovered phase. However, *Bdkrb1* was significantly upregulated in all mice groups following *C. rodentium* infection, though TLR9 KO group alone showed persistent high expression levels of *Bdkrb1* in the recovered phase (**Figures 5A, B**; **Supplementary Figure S9**). These observations are consistent with findings noted in a previous report described above (46), and also indicate that an increase in *Bdkrb2* expression occurs during recovery from infection and *Bdkrb1* levels are not downregulated in susceptible hosts. Moreover, FMT-treated TLR9 KO mice exhibited higher *Bdkrb2* but not *Bdkrb1* levels compared with FMT-treated WT mice (**Supplementary Figure 8B**), indicating that dysbiosis can, at least in part, increase *Bdkrb2*, though only in susceptible hosts. Next, immunofluorescence staining was used to determine localization of bradykinin receptors in the intestine. Bdkrb1 and Bdkrb2 were found to be predominantly expressed in mucosal epithelium, but not the enteric nervous system. Notably, Bdkrb2 exhibited a much greater intensity in infected TLR9 KO as compared to infected WT mice (**Figure 6**). These findings indicate that *C. rodentium* can induce persistent upregulation of bradykinin receptors in colon epithelium in the absence of TLR9, which might be one of the mechanisms related to development of PI-IBS noted in the present mice.

**Table 1:** Pathway analysis.

Pathway	Upregulated genes	Downregulated genes	p value
Striated muscle contraction		*Tnnt2, Myh1, Mybpc1, Myh8, Myom1, Myl9*	0.000079
Adar1 editing deficiency immune response	*Oasl2, Rsad2, Slfn4, Zbp1, Ifit1, Ddx60*	*Nfkbia*	0.000156
Chemokine signaling pathway	*Cxcr4, Cxcr5*	*Ppbp, Prkcb, Plcb4, Cxcl5*, *Ccl21c, Nfkbia, Ccl21a*	0.003339
Retinol metabolism	*Sult1a1, Rbp2*	*Aldh1a3, Rbp1*	0.003425
Urea cycle and metabolism of amino groups		*Arg1, Acy1, Ckm*	0.003793
SRF and miRs in smooth muscle differentiation and proliferation		*Mir143, Myocd*	0.023374
Peptide GPCRs	*Bdkrb2, Cxcr4*	*Npy6r, Tacr2*	0.025956
B cell receptor signaling pathway	*Ptprc, Bank1, Cr2*	*Prkcb, Atp2b4, Nfkbia*	0.040079

Mouse Gene ST (Filgen, Aichi, Japan) assays were performed with colonic samples from *C. rodentium*-treated Toll-like receptor (TLR)9 knockout (KO) mice and wild-type (WT) mice. Based on the results of altered gene expression, pathway analysis was additionally performed using a Microarray Data Analysis Tool Ver. 3.2 software (Filgen) to classify the data into functional subgroups. Up- and down-regulated genes with a p value less than 0.05 are listed.

### 3.5 Therapeutic efficacy of selective bradykinin B1/B2 receptor antagonists for PI-IBS

**Figure 7:**
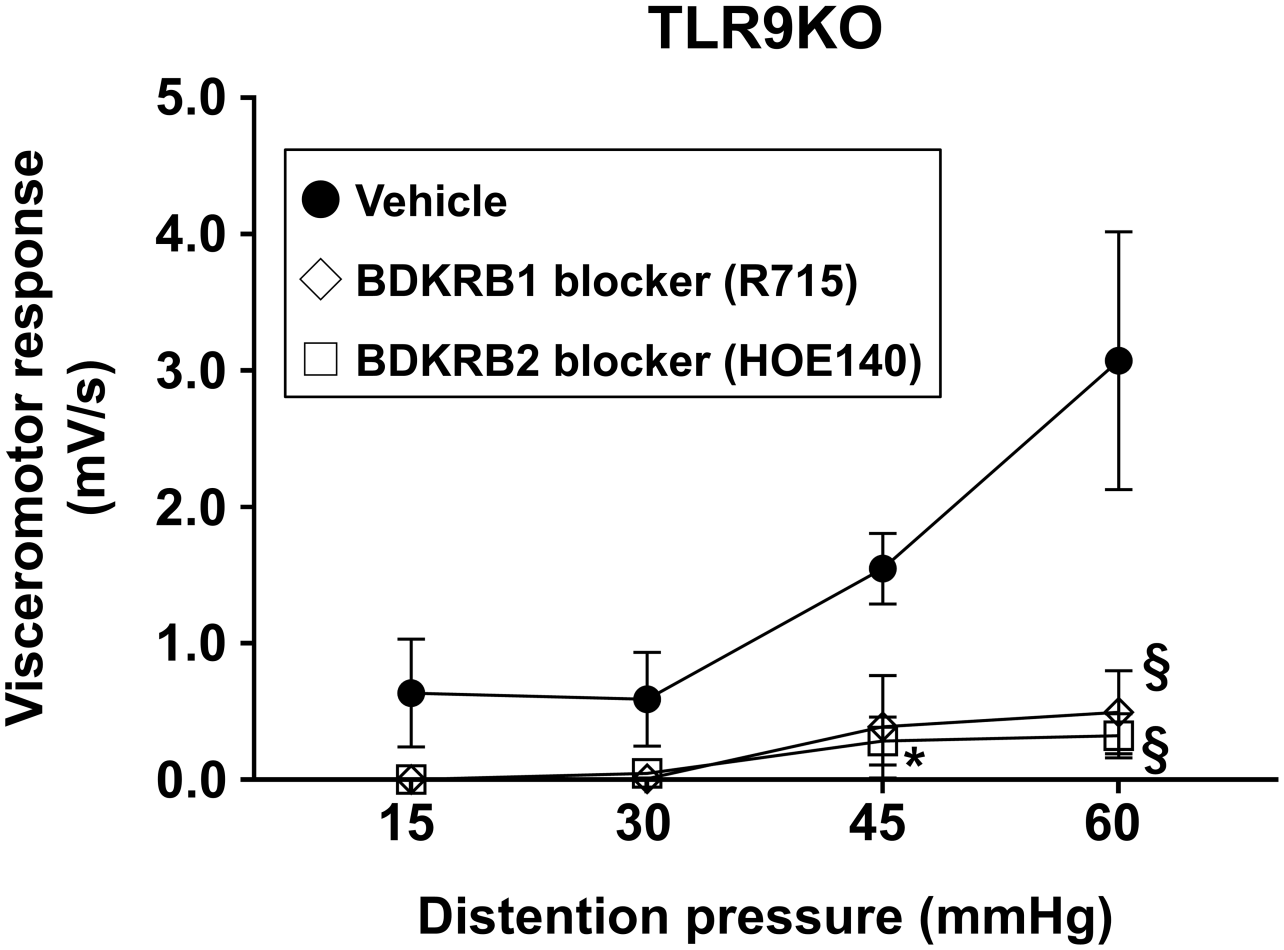
Neutralizing Bdkrb1 and Bdkrb2 ameliorate visceral hypersensitivity. R715 (1 mg/kg), HOE 140 (1 mg/kg), or the vehicle was intraperitoneally administered into *C. rodentium*-treated TLR9 KO mice (n=3/group) at six weeks after infection. VMR to colorectal distention was evaluated using rectal balloon dilation at two hours after treatment with R715 or HOE 140. Values were obtained using a two-way ANOVA test and are presented as the mean ± SEM. *p <0.05, §p <0.0001, as compared with control group. Tukey’s multiple comparisons test was used for *post hoc* analysis.

Based on the results obtained showing increased expression of intestinal bradykinin receptors in *C. rodentium*-treated TLR9 KO mice, the effects of R715 and HOE 140, selective antagonists of Bdkrb1 and 2, respectively, for treatment of PI-IBS were examined. HOE 140 has a similar affinity to bradykinin and is clinically used for a type of hereditary angioedema (47–50). Two hours prior to evaluation of VMR to colorectal distention, each agent was separately administered intraperitoneally into *C. rodentium*-treated TLR9 KO mice (1 mg/kg). Interestingly, a single injection of HOE 140 was sufficient to attenuate visceral hyperalgesia in mice affected by PI-IBS (**Figure 7**). Furthermore, R715 also showed an effect on visceral hyperalgesia (**Figure 7**). It is thus considered that an antagonist of bradykinin receptors can alleviate abdominal symptoms in cases of PI-IBS with a TLR9 signaling defect.

## 4 Discussion

Following acute gastroenteritis caused by a viral, bacterial, or parasitic infection, 3.6% to 31.6% of affected individuals develop PI-IBS (13, 14, 41, 51–57). Common features of PI-IBS include chronic abdominal pain, abnormal bowel movements, and bloating, though the severity and dominant phenotype of IBS symptoms largely differ among individuals. It also remains unclear why only certain populations develop PI-IBS after an intestinal infection. Previous genome-wide association study results from investigation of a waterborne outbreak (19) inspired us to focus on TLR9 signaling as part of the pathogenesis of PI-IBS. The present results revealed development of persistent visceral hyperalgesia in TLR9 KO mice following *C. rodentium* infection, despite the absence of prolonged mucosal inflammation or intestinal hyperpermeability. In addition, they suggest that upregulation of bradykinin receptors, especially Bdkrb2, is a key factor for development of PI-IBS, indicating its potential as a target of therapy. Furthermore, alterations in gut microbiota may also be involved, at least in part, in the pathogenesis of PI-IBS in a TLR9-deficient state.

TLR9 is an innate immune receptor that recognizes bacterial CpG-DNA, known to modulate immune responses. Human studies have shown that genetic polymorphisms in TLR9, rs352139 and rs5743836, are associated with PI-IBS (19), though their precise impact on TLR9 signaling remains unclear. The present results indicate that these SNPs may play a pathogenic role in PI-IBS development because of weakened or diminished TLR9 signaling. Nevertheless, conflicting findings noted in studies of autoimmune diseases such as systemic lupus erythematosus (58–61) have implicated both excessive and attenuated TLR9 signaling levels, thus further investigations are needed to clarify its precise role in PI-IBS susceptibility.

A major challenge is lack of an animal model that fully replicates human PI-IBS, as gut microbiota, mucosal immunity, diet, and psychological factors differ among species (62). *Escherichia coli* and *Campylobacter jejuni* are pathogens that have been linked to PI-IBS in humans by causing severe colitis with persistent mucosal hyperpermeability (35). *C. rodentium*, a murine pathogen biologically similar to human enteropathogenic *Escherichia coli* (36), was chosen for the present study, as it induces non-lethal acute colitis in mouse strains such as C57BL/6 and BALB/c (40, 63). Although previous studies have reported that *C. rodentium* induced visceral hyperalgesia in normal mice (64–66), those evaluations were conducted at early time points when mucosal inflammation likely persisted. In the present study, IBS features were assessed at six weeks following infection, a time point considered sufficient for complete recovery from colitis, thus the findings are considered more appropriate for evaluation of PI-IBS.

As for the mechanism of PI-IBS, persistent low-grade mucosal inflammation with increased intestinal permeability after improvement of infection has been reported to contribute to its features (39, 40, 65). In clinical practice, enterocolitis severity is thought to be associated with development of PI-IBS (15–17, 67, 68). However, the present findings indicated that visceral hyperalgesia in *C. rodentium*-infected TLR9 KO mice was not associated with the severity of acute colitis, residual mucosal inflammation, or sustained barrier dysfunction, while WT mice with more severe inflammation did not develop PI-IBS at six weeks after the initial infection. Mondelaers et al. reported that visceral hypersensitivity induced by *C. rodentium* in Th1-predominant C57BL/6 WT mice was transient and limited to the acute phase, whereas Th2-predominant Balb/c mice retained visceral hypersensitivity up to a later time point despite similar acute gastroenteritis severity (66). Those findings suggest that a Th2-skewed immune background may predispose individuals to PI-IBS. TLR9 is a key regulator of the innate immune system, and promotes Th1 differentiation through IL-12 and IFN-γ production (69). Given the role of TLR9 in shaping Th1/Th2 balance, its absence may lead to a relative Th2 predominance, which could contribute to the sustained visceral hypersensitivity observed in Balb/c mice. However, our microarray dataset at the recovery phase did not reveal significant differences between infected WT and TLR9KO mice in Th1- or Th2-specific pathways (data not shown), although further analysis focusing on specific immune profiles is warranted. Clinical studies have also indicated that individuals with a Th2-dominant immune profile have increased risk of developing PI-IBS. A prospective study reported that patients with a Th2-skewed immune response had a significantly higher likelihood of developing PI-IBS one year after an episode of infectious gastroenteritis (70). It is thus suggested that a Th2-dominant immune dysfunction may contribute not only to features of intestinal inflammation but also persistent visceral hypersensitivity, which may link innate immune dysregulation to PI-IBS susceptibility and should be addressed in future studies. The present findings indicate that TLR9, rather than other TLRs, has a specific role in PI-IBS pathogenesis, while its absence may be one of the risk factors for PI-IBS development in humans.

In addition, gastrointestinal microbiota alterations (dysbiosis) are frequently seen in IBS patients (39). A previous study found that *Bacteroidetes* phylum was abundant in PI-IBS patients (71), while the present findings showed that the proportion of *Bacteroidetes* in *C. rodentium*-infected TLR9 KO mice was similar to that in the other groups. Instead, *Clostridium_sensu_stricto* was increased in TLR9 KO mice following *C. rodentium* infection. Li et al. demonstrated that this genus was enriched in stool samples from chemically induced post-inflammatory IBS model rats (60), suggesting a potential link to visceral hypersensitivity. However, a recent study of PI-IBS in humans that develops following a *Campylobacter* infection showed distinct microbiota changes characterized by reduced levels of *Clostridiales* and *Ruminococcaceae*, along with increased *Proteobacteria*, *Fusobacteria*, and *Gammaproteobacteria* levels (72). These different findings highlight the impact of host species, infection type, and immune background on microbiota composition. Although dysbiosis has often been implicated in the pathogenesis of PI-IBS, the present FMT results suggest that microbiota changes alone are insufficient to drive PI-IBS-like symptoms together with Bdkrb upregulation, as antibiotic-treated WT mice did not exhibit visceral hypersensitivity after FMT with feces from TLR9 KO mice with PI-IBS like symptoms. Therefore, it is likely that additional genetic factors, such as immune response to an intestinal infection or neuroinflammatory pathways, contribute to development of IBS symptoms.

Microarray analysis identified bradykinin receptors as potential mediators of PI-IBS in the TLR9 KO mice. Bradykinin is a well-established mediator of pain and inflammation that acts through two receptors; Bdkrb1, induced during inflammation, and Bdkrb2, which is homeostatically expressed (46). The present results showed the presence of Bdkrb1/2 in intestinal epithelial cells but not the enteric nervous system, which was confirmed by immunohistochemistry findings, and also attenuation of visceral hyperalgesia in TLR9 KO mice by selective inhibition of Bdkrb1/2. It is thus considered that epithelial changes, rather than neuronal alterations, may drive visceral hypersensitivity. HOE 140, a selective Bdkrb2 antagonist, is clinically used as a pharmaceutical agent for treatment of acute attacks of hereditary angioedema, as it can effectively reduce pain and swelling. Given this pharmacological profile, HOE 140 is considered more appropriate for managing acute pain episodes rather than for prophylactic treatment of IBS-induced pain. Thus, in PI-IBS cases it is expected to be therapeutically beneficial for relief of symptoms rather than prevention of onset of visceral hypersensitivity.

Recent studies have suggested that TLR signaling may play a role in modulating bradykinin receptor expression. While the specific mechanism by which TLR9 deficiency increases Bdkrb1/2 expression remains unclear, prior research has found that TLR2 activation upregulates bradykinin receptor expression via NF-κB and MAPK signaling pathways (73). Given that TLR9 and TLR2 share overlapping downstream signaling cascades, it is plausible that TLR9 deficiency could indirectly influence bradykinin receptor expression through compensatory mechanisms involving other TLRs. Additional investigations will be needed to determine whether TLR9 directly modulates bradykinin receptor expression or if other innate immune pathways contribute to this phenomenon.

This study has several limitations. First, tests using alternative models, such as with a viral or protozoal enteric infection, were not conducted due to the constraints of our animal facility. Second, the precise molecular mechanism linking a defect of TLR9 to Bdkrb2 upregulation remains unclear. As TLR9 is not present in intestinal epithelium (74) where Bdkrb2 is predominantly present, the possibility of cell-intrinsic regulation of Bdkrb2 through TLR9 activation would be low. Further examinations will be required to elucidate its role in PI-IBS pathogenesis. Third, we cannot definitively localize the site of bradykinin pathway modulation. Although BDKRB1/2 expression increased predominantly in the colonic epithelium, contributions from primary sensory neurons/dorsal root ganglia (DRG) or spinal circuits cannot be excluded, and our systemic antagonist experiments do not rule out site- specific effects. Future studies are required to define the locus of action.

In conclusion, results obtained in the present study led to identification of TLR9 as a critical regulator in PI-IBS development, with Bdkrb2 upregulation also found in the presence of pathobionts. Notably, findings indicating that Bdkrb1/2 antagonism ameliorates symptoms suggest a potential therapeutic avenue for PI-IBS treatment. Future studies are needed explore the broader spectrum of microbial and immune interactions contributing to PI-IBS pathogenesis, as well as the clinical applicability of targeting the bradykinin pathway for symptom relief.

## Data Availability

The microarray data generated in this study have been deposited in the DDBJ Genomic Expression Archive (GEA) under the accession number E-GEAD-1156. The 16S rRNA sequencing data are publicly available via the DDBJ BioProject database under BioProject accession number PRJDB37949. Associated metadata and sequence files can be accessed through the DDBJ website at: https://ddbj.nig.ac.jp.

## Supplementary material

Supplementary Figure 1 *C. rodentium* did not induce intestinal permeability in acute phase of infection. Fluorescein isothiocyanate (FITC)-dextran with a molecular weight of 4 kDa was administered to WT, TLR2 KO, TLR4 KO, and TLR9 KO mice with or without *C. rodentium* infection by gavage at two weeks after infection (n=6/group). Blood was obtained three hours after administration and the FITC-dextran concentration determined. Values were obtained with a one-way ANOVA test and are presented as the mean. N.S. indicates not statistically significant.

Supplementary Figure 2 Electromyography of *C. rodentium*-treated TLR9 KO mice. Representative images showing VMR with 60-mmHg rectal balloon dilation. Left: *C. rodentium*-treated WT mouse, right: *C. rodentium*-treated TLR9 KO mouse.

Supplementary Figure 3 No significant gender differences in VMR to colorectal distention. *C. rodentium* or PBS was administered to WT, TLR2 KO, TLR4 KO, and TLR9 KO mice (6 males, 6 females) on day 1. Five weeks after infection, the mice were anesthetized and electrodes implanted in the abdominal wall, then evaluation of VMR to colorectal distention was performed at six weeks after infection. Four different levels of pressure (15, 30, 45, 60 mmHg) were used for balloon dilation in each mouse. A 10-second distention was performed three times with one-minute intervals at each pressure level and the median value determined. Values were obtained using a two-way ANOVA test and are presented as the mean ± SEM. *p <0.05, ^§^p <0.0001, as compared with PBS group. Tukey’s multiple comparisons test was used for *post hoc* analysis.

Supplementary Figure 4 *C. rodentium* did not induce intestinal permeability in recovered phase. FITC-dextran with a molecular weight of 4 kDa was administered to WT, TLR2 KO, TLR4 KO, and TLR9 KO mice with or without *C. rodentium* infection by gavage at six weeks after infection (n=6/group). Blood was obtained three hours after administration and the FITC-dextran concentration determined. Values were obtained with a one-way ANOVA test and are presented as the mean. N.S. indicates not statistically significant.

Supplementary Figure 5 Proportion of intestinal microbiota at phylum level. Cecal contents were obtained from WT, TLR2 KO, TLR4 KO, and TLR9 KO mice with *C. rodentium* infection after six weeks. Bacterial DNA was extracted and 16S rRNA assays were performed. Proportions of intestinal microbiota at the phylum level are shown.

Supplementary Figure 6 *Clostridium_sensu_stricto* increased in *C. rodentium*-infected TLR9 KO mice. Cecal contents were obtained from WT, TLR2 KO, TLR4 KO, and TLR9 KO mice at six weeks after *C. rodentium* infection (n=3/group). Bacterial DNA was then extracted and 16S rRNA testing performed. Shown are bacterial proportions at the **(A)** family and **(B)** genus level. **(C)***Clostridiaceae_1* abundance at family level (WT vs. TLR2 KO vs. TLR4 KO vs. TLR9 KO; 0.76%, 0.93%, 2.46%, 4.18%, respectively). **(D)***Clostridium_sensu_stricto* abundance at genus level (WT vs. TLR2 KO vs. TLR4 KO vs. TLR9 KO; 0.75%, 0.92%, 2.45%, 4.18%, respectively). Values were obtained with a one-way ANOVA test and are presented as the mean.

Supplementary Figure 7 Experimental procedures for determination of visceral hypersensitivity in mice following fecal microbiota transplantation (FMT). Preexisting gut microbiota in recipient mice was depleted by a three-day treatment with a broad-spectrum antibiotic cocktail (oral gavage: vancomycin and metronidazole, drinking water: ampicillin and neomycin). One day after antibiotic washout, recipients were administered donor microbiota from post-infectious TLR9 KO mice by oral gavage twice. Electrode implantation and visceral sensitivity assessment using a barostat were performed at four and five weeks, respectively, after FMT.

Supplementary Figure 8 Visceral hypersensitivity was not induced by fecal microbiota transplantation (FMT) from PI-IBS mice. **(A)** Following FMT with feces obtained from post-infectious TLR9 KO mice, visceral sensitivity was assessed using a barostat in the WT and TLR9 KO groups (n=6/group). Values were analyzed by two-way ANOVA and are presented as mean ± SEM. **(B)** Expression levels of *Bdkrb1* and *Bdkrb2* in distal colons from WT and TLR9 KO mice with FMT (n=6/group) were assessed by RT-PCR. Values were analyzed using Student’s t-test and are presented as mean ± SEM. *p <0.05, as compared with WT mice.

Supplementary Figure 9 Bdkrb1/2 profiles of *C. rodentium*-infected mice in acute phase. *C. rodentium* or PBS was administered to WT, TLR2 KO, TLR4 KO, and TLR9 KO mice (n=6/group). Expression levels of Bdkrb1 and Bdkrb2 in distal colons were assessed by RT-PCR. Values were obtained with a one-way ANOVA test and are presented as the mean. *p <0.05, ‡p <0.001, §p <0.0001, N.S., not significant as compared with PBS group. Holm-Sidak’s multiple comparisons test was used for *post hoc* analysis.
