# Supplementary figures and images for: Association between Toll-like receptor 9 signaling defect and developing post-infectious irritable bowel syndrome
Source: Front Immunol. 2025 Nov 10;16:1672117. doi: 10.3389/fimmu.2025.1672117 (PMC12642955; doi:10.3389/fimmu.2025.1672117)

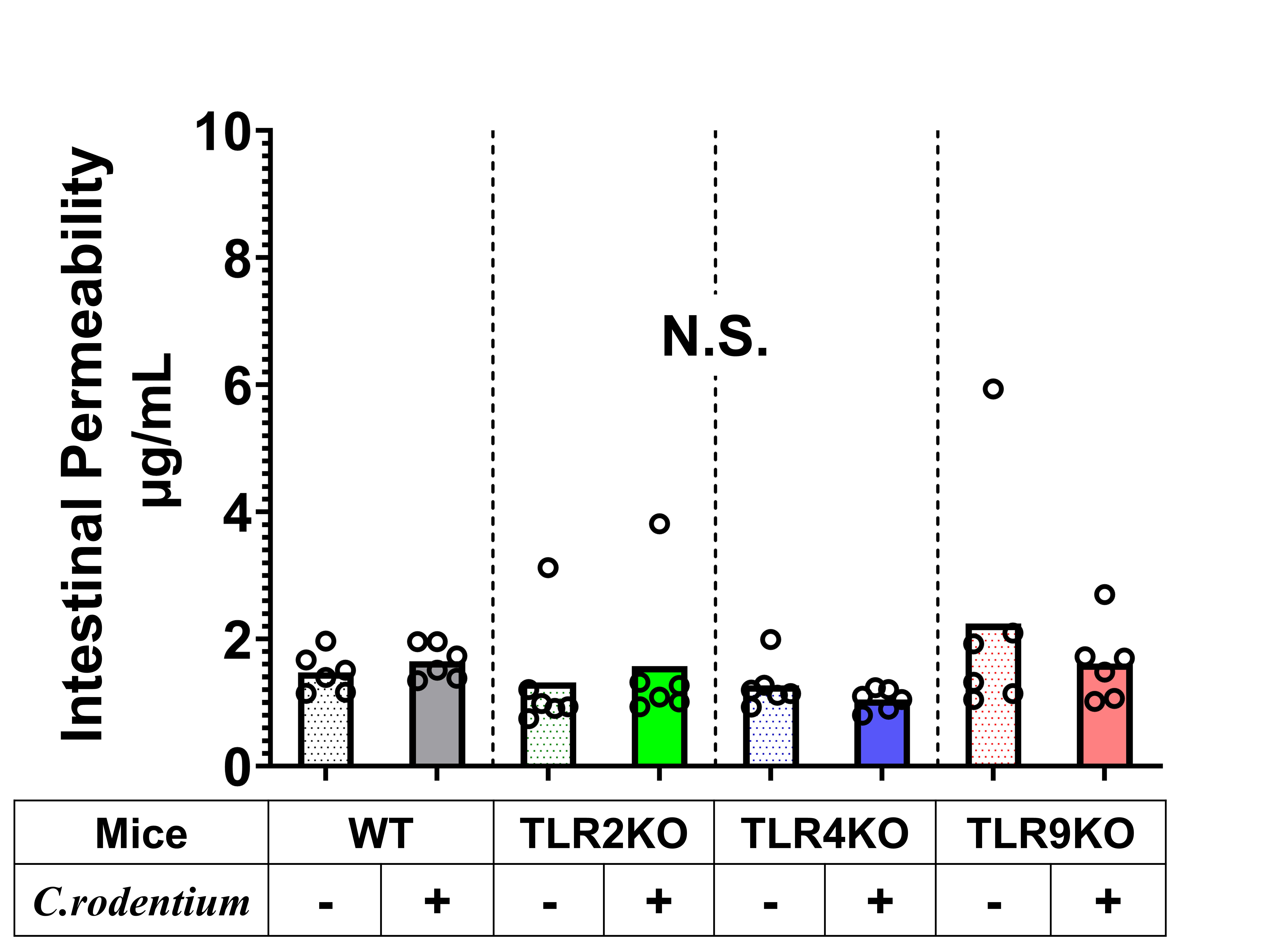

Supplement: Supplementary Figure 1 — C. rodentium did not induce intestinal permeability in acute phase of infection. Fluorescein isothiocyanate (FITC)-dextran with a molecular weight of 4 kDa was administered to WT, TLR2 KO, TLR4 KO, and TLR9 KO mice with or without C. rodentium infection by gavage at two weeks after infection (n=6/group). Blood was obtained three hours after administration and the FITC-dextran concentration determined. Values were obtained with a one-way ANOVA test and are presented as the mean. N.S. indicates not statistically significant. [file Image1.tif]

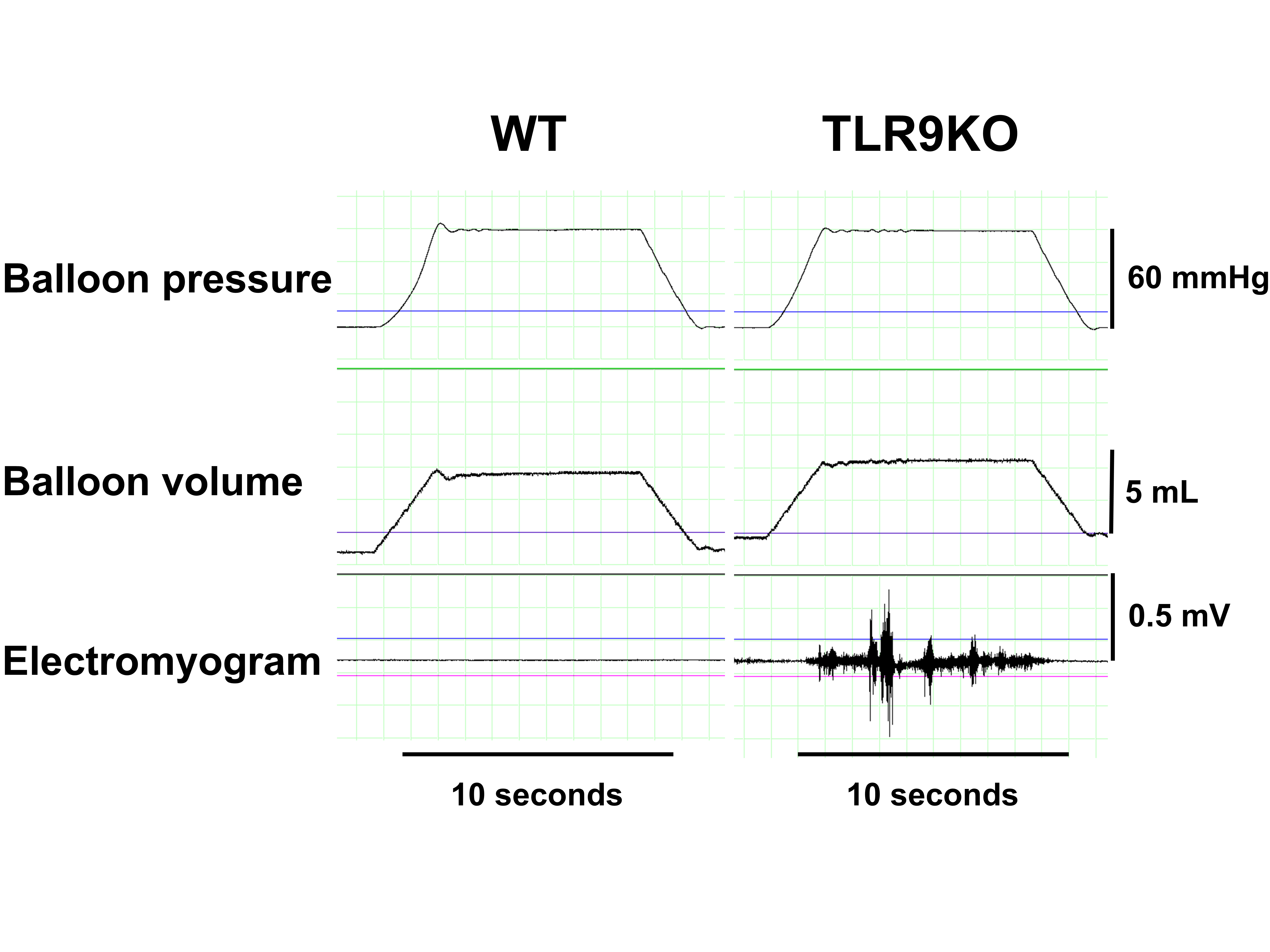

Supplement: Supplementary Figure 2 — Electromyography of C. rodentium-treated TLR9 KO mice. Representative images showing VMR with 60-mmHg rectal balloon dilation. Left: C. rodentium-treated WT mouse, right: C. rodentium-treated TLR9 KO mouse. [file Image2.tif]

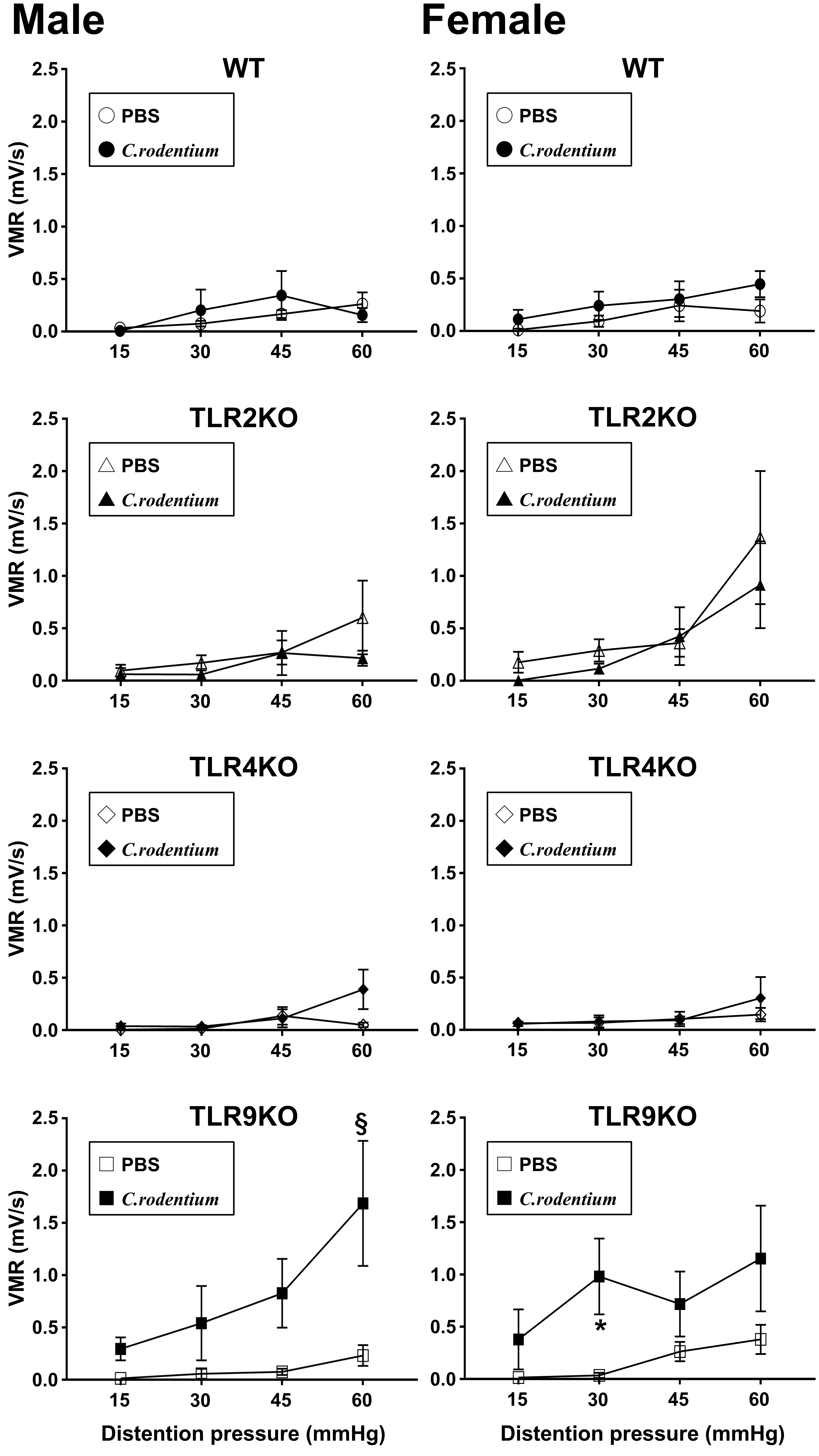

Supplement: Supplementary Figure 3 — No significant gender differences in VMR to colorectal distention. C. rodentium or PBS was administered to WT, TLR2 KO, TLR4 KO, and TLR9 KO mice (6 males, 6 females) on day 1. Five weeks after infection, the mice were anesthetized and electrodes implanted in the abdominal wall, then evaluation of VMR to colorectal distention was performed at six weeks after infection. Four different levels of pressure (15, 30, 45, 60 mmHg) were used for balloon dilation in each mouse. A 10-second distention was performed three times with one-minute intervals at each pressure level and the median value determined. Values were obtained using a two-way ANOVA test and are presented as the mean ± SEM. *p <0.05, §p <0.0001, as compared with PBS group. Tukey’s multiple comparisons test was used for post hoc analysis. [file Image3.tif]

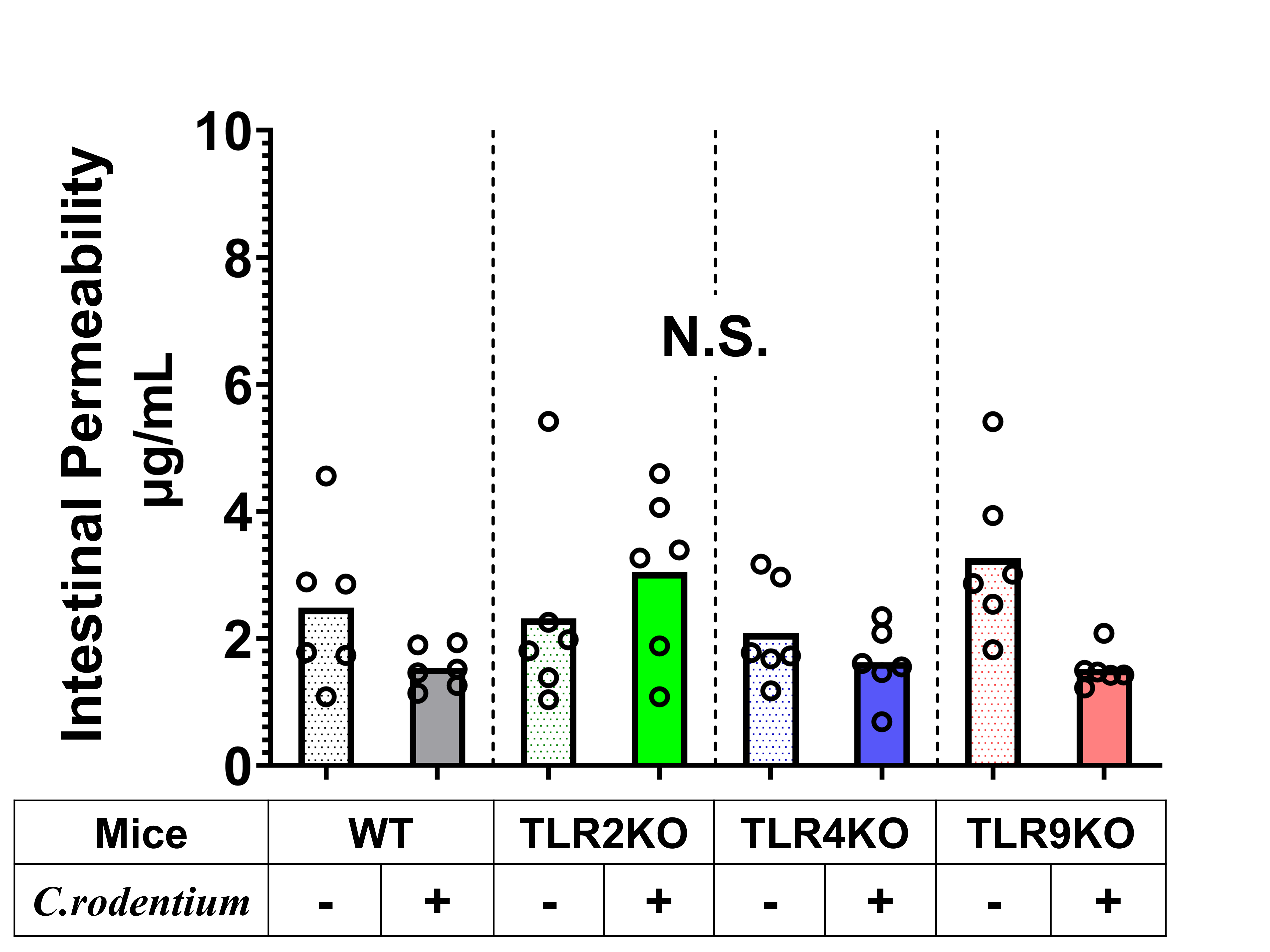

Supplement: Supplementary Figure 4 — C. rodentium did not induce intestinal permeability in recovered phase. FITC-dextran with a molecular weight of 4 kDa was administered to WT, TLR2 KO, TLR4 KO, and TLR9 KO mice with or without C. rodentium infection by gavage at six weeks after infection (n=6/group). Blood was obtained three hours after administration and the FITC-dextran concentration determined. Values were obtained with a one-way ANOVA test and are presented as the mean. N.S. indicates not statistically significant. [file Image4.tif]

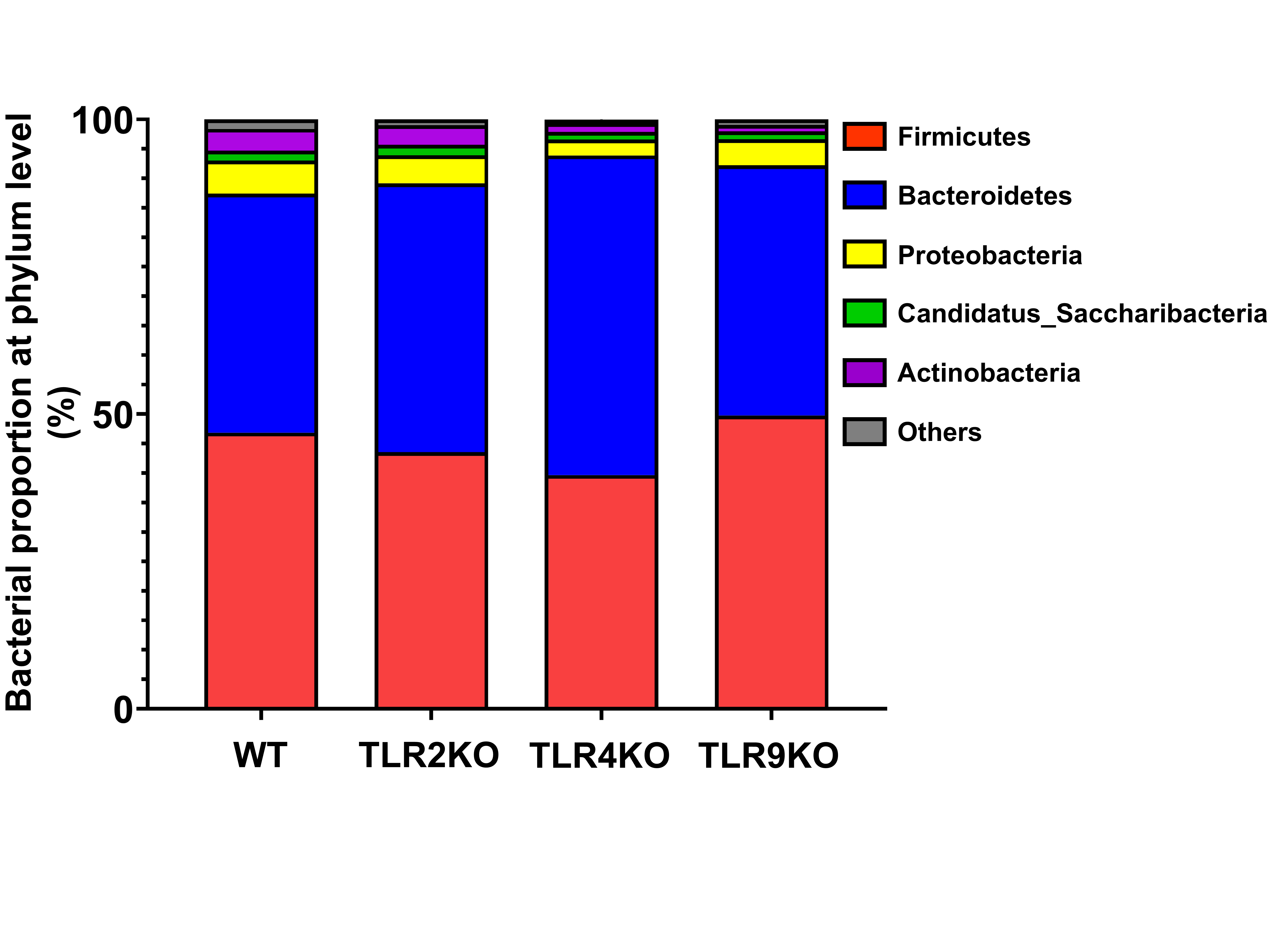

Supplement: Supplementary Figure 5 — Proportion of intestinal microbiota at phylum level. Cecal contents were obtained from WT, TLR2 KO, TLR4 KO, and TLR9 KO mice with C. rodentium infection after six weeks. Bacterial DNA was extracted and 16S rRNA assays were performed. Proportions of intestinal microbiota at the phylum level are shown. [file Image5.tif]

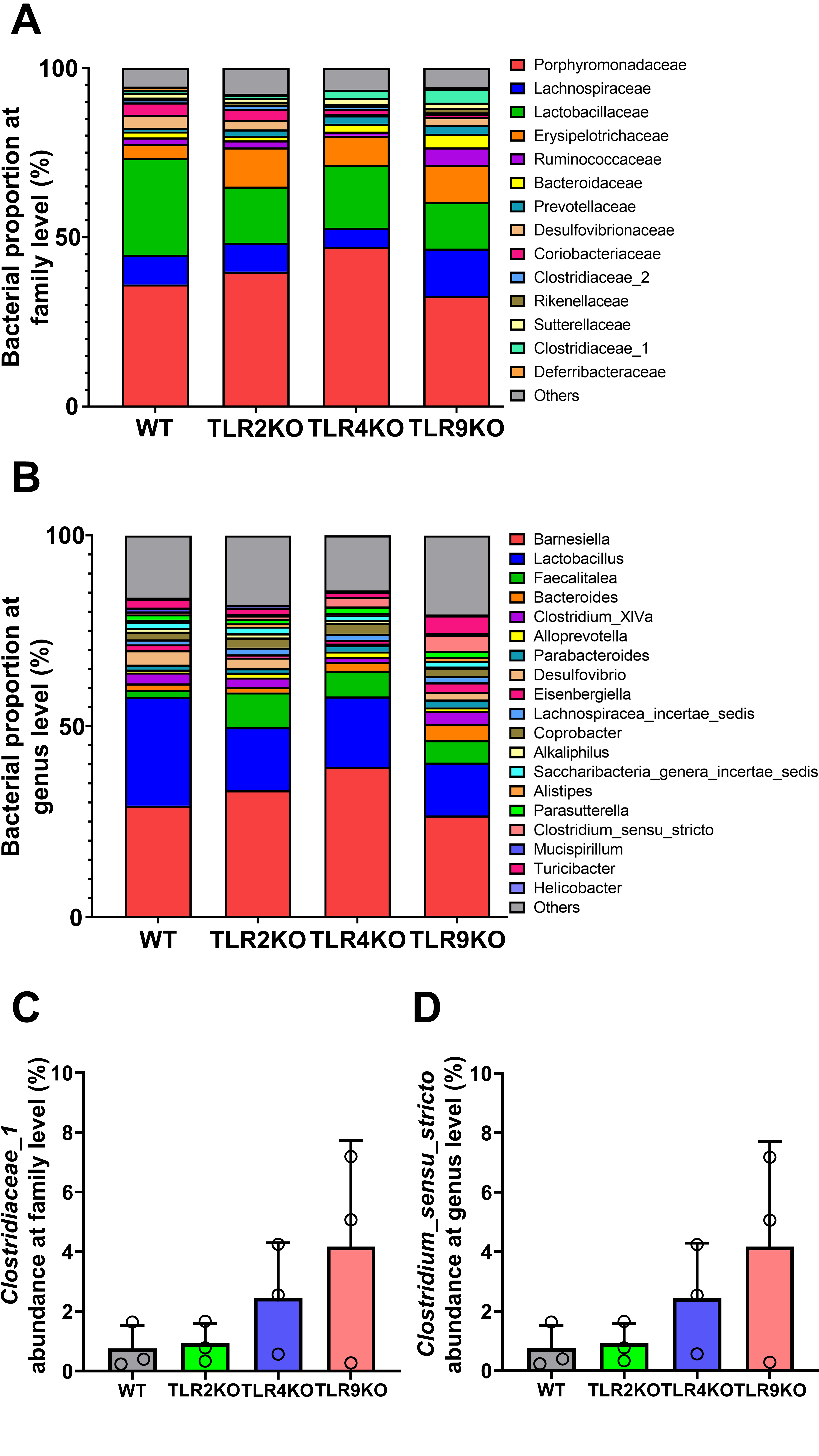

Supplement: Supplementary Figure 6 — Clostridium_sensu_stricto increased in C. rodentium-infected TLR9 KO mice. Cecal contents were obtained from WT, TLR2 KO, TLR4 KO, and TLR9 KO mice at six weeks after C. rodentium infection (n=3/group). Bacterial DNA was then extracted and 16S rRNA testing performed. Shown are bacterial proportions at the (A) family and (B) genus level. (C)Clostridiaceae_1 abundance at family level (WT vs. TLR2 KO vs. TLR4 KO vs. TLR9 KO; 0.76%, 0.93%, 2.46%, 4.18%, respectively). (D)Clostridium_sensu_stricto abundance at genus level (WT vs. TLR2 KO vs. TLR4 KO vs. TLR9 KO; 0.75%, 0.92%, 2.45%, 4.18%, respectively). Values were obtained with a one-way ANOVA test and are presented as the mean. [file Image6.tif]

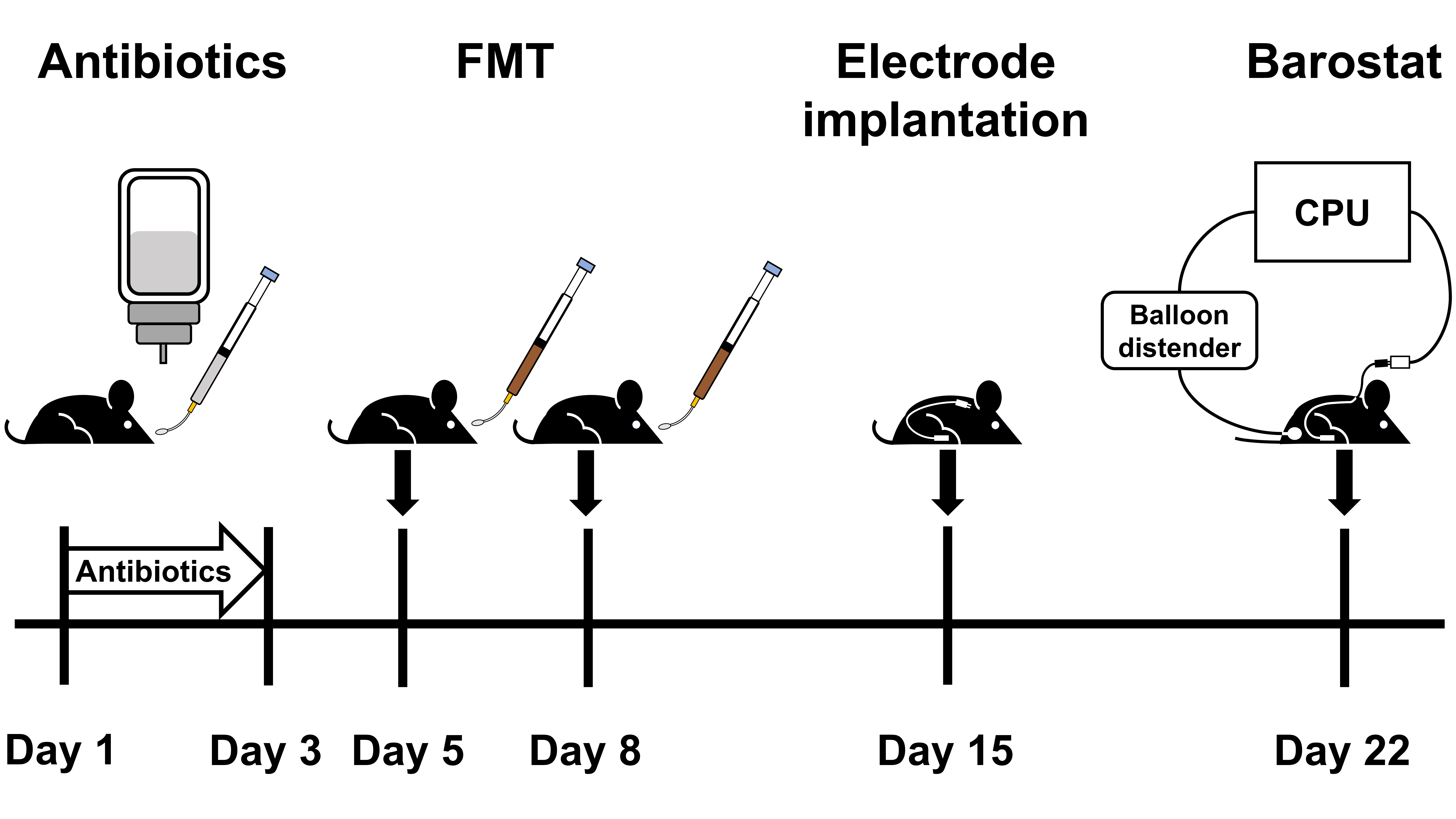

Supplement: Supplementary Figure 7 — Experimental procedures for determination of visceral hypersensitivity in mice following fecal microbiota transplantation (FMT). Preexisting gut microbiota in recipient mice was depleted by a three-day treatment with a broad-spectrum antibiotic cocktail (oral gavage: vancomycin and metronidazole, drinking water: ampicillin and neomycin). One day after antibiotic washout, recipients were administered donor microbiota from post-infectious TLR9 KO mice by oral gavage twice. Electrode implantation and visceral sensitivity assessment using a barostat were performed at four and five weeks, respectively, after FMT. [file Image7.tif]

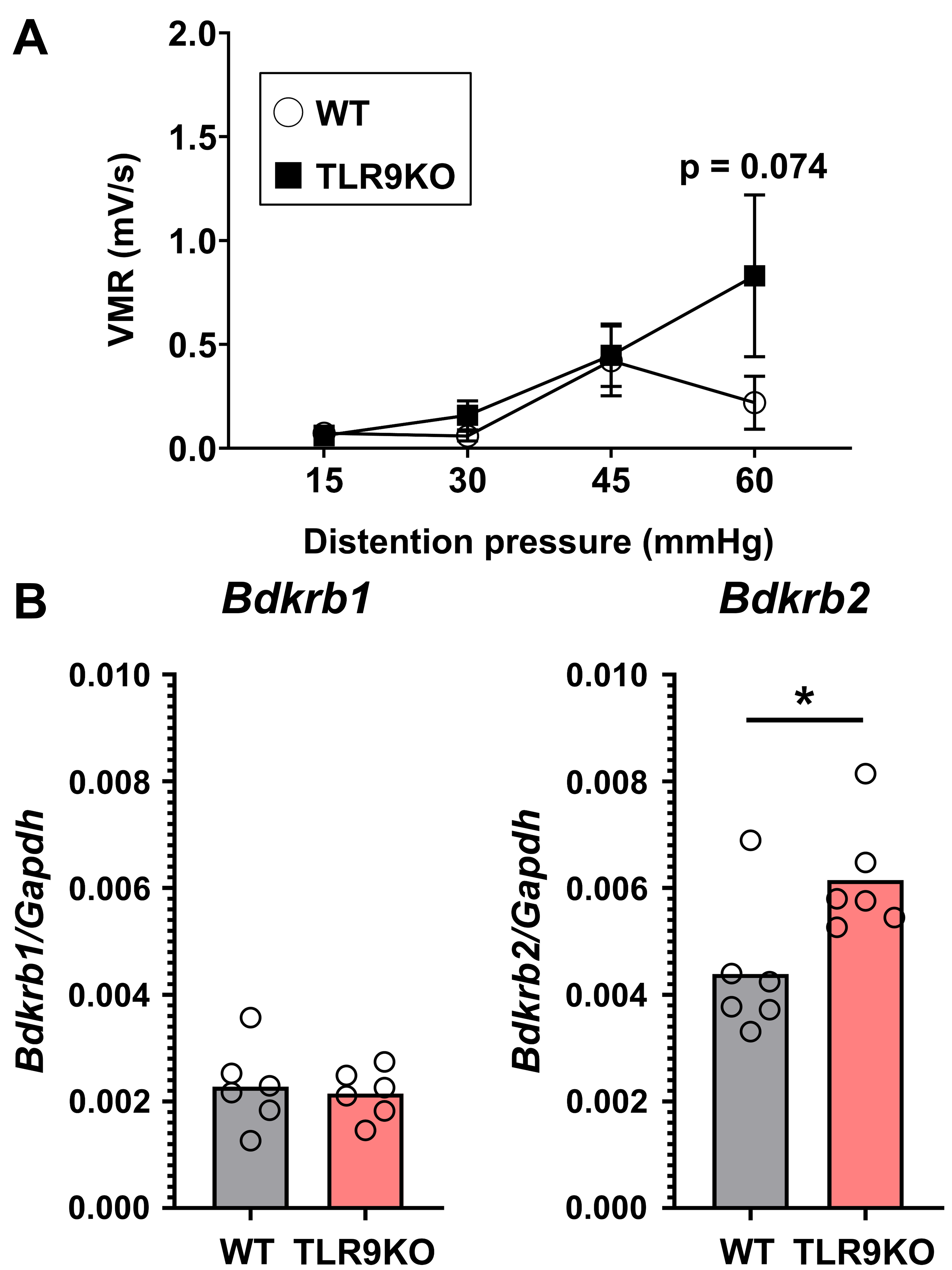

Supplement: Supplementary Figure 8 — Visceral hypersensitivity was not induced by fecal microbiota transplantation (FMT) from PI-IBS mice. (A) Following FMT with feces obtained from post-infectious TLR9 KO mice, visceral sensitivity was assessed using a barostat in the WT and TLR9 KO groups (n=6/group). Values were analyzed by two-way ANOVA and are presented as mean ± SEM. (B) Expression levels of Bdkrb1 and Bdkrb2 in distal colons from WT and TLR9 KO mice with FMT (n=6/group) were assessed by RT-PCR. Values were analyzed using Student’s t-test and are presented as mean ± SEM. *p <0.05, as compared with WT mice. [file Image8.tif]

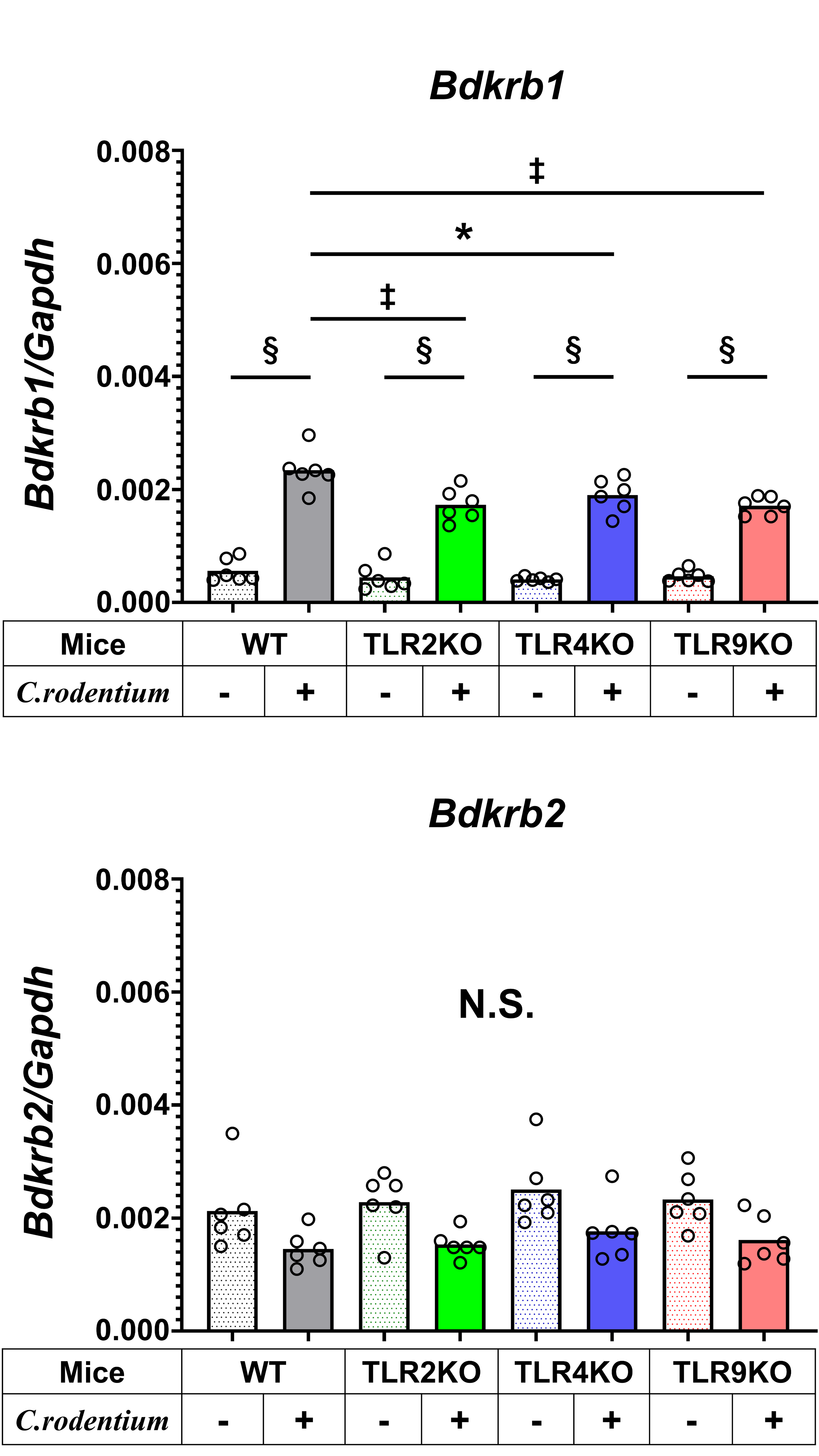

Supplement: Supplementary Figure 9 — Bdkrb1/2 profiles of C. rodentium-infected mice in acute phase. C. rodentium or PBS was administered to WT, TLR2 KO, TLR4 KO, and TLR9 KO mice (n=6/group). Expression levels of Bdkrb1 and Bdkrb2 in distal colons were assessed by RT-PCR. Values were obtained with a one-way ANOVA test and are presented as the mean. *p <0.05, ‡p <0.001, §p <0.0001, N.S., not significant as compared with PBS group. Holm-Sidak’s multiple comparisons test was used for post hoc analysis. [file Image9.tif]
